# Mixture Multilevel SEM vs. Multilevel SEM for comparing structural relations across groups in presence of measurement non-invariance

**DOI:** 10.3389/fpsyg.2025.1463790

**Published:** 2025-07-28

**Authors:** Hongwei Zhao, Jeroen K. Vermunt, Kim De Roover

**Affiliations:** ^1^Quantitative Psychology and Individual Differences, KU Leuven, Leuven, Belgium; ^2^Department of Methodology and Statistics, Tilburg University, Tilburg, Netherlands

**Keywords:** structural equation modeling (SEM), measurement invariance (MI), multilevel modeling, mixture modeling, multielvel SEM

## Abstract

Structural equation modeling (SEM) is commonly used to explore relations between latent variables, such as beliefs and attitudes. However, comparing structural relations across a large number of groups, such as countries or classrooms, can be challenging. Existing SEM approaches may fall short, especially when measurement non-invariance is present. In this paper, we propose Mixture Multilevel SEM (MixML-SEM), a novel approach to comparing relationships between latent variables across many groups. MixML-SEM gathers groups with the same structural relations in a cluster, while accounting for measurement non-invariance in a parsimonious way by means of random effects. Specifically, MixML-SEM captures measurement non-invariance using multilevel confirmatory factor analysis and, then, it estimates the structural relations and mixture clustering of the groups by means of the structural-after-measurement approach. In this way, MixML-SEM ensures that the clustering is focused on structural relations and unaffected by differences in measurement. In contrast, Multilevel SEM (ML-SEM) estimates measurement and structural models simultaneously, and both with random effects. In comparison to ML-SEM, MixML-SEM provides better estimates of the structural relations, especially when (some of) the groups are large. This is because combining information from multiple groups within a cluster leads to more accurate estimates of the structural relations, whereas, in case of ML-SEM, these estimates are affected by shrinkage bias. We demonstrate the advantages of MixML-SEM through simulations and an empirical example on how social pressure to be happy relates to life satisfaction across 40 countries.

## Introduction

Social science research often aims to gain insight into complex human behavior by studying the relations between constructs (e.g., satisfaction, emotions), often quantified by regression coefficients. Comparing these relations across groups helps reveal how they differ across populations, cultures, or contexts. For instance, Kuppens et al. (2008) explored the association between life satisfaction and positive and negative emotions across 46 countries, while Pakarinen et al. (2020) investigated how emotional support from teachers related to the development of social competence in children across 47 preschool classrooms.

Structural Equation Modeling (SEM; Bollen, 1989; Hoyle, 2012) is the state-of-the-art technique for analyzing relations among several constructs, referred to as “structural relations” in this framework. In studies involving multiple groups, Multigroup SEM allows researchers to estimate a SEM model for all groups and test whether the structural relations are consistent across groups. When many groups are involved, differences in structural relations are more likely to emerge. Conducting pairwise comparisons of the group-specific relations can help identify group differences and similarities, but this quickly becomes infeasible as the number of groups increases (e.g., 1,035 pairwise comparisons for 46 groups). Multilevel SEM (ML-SEM) can parsimoniously capture differences in structural relations across many groups by means of normally distributed random effects, but this does not help in achieving our primary goal of pinpointing which groups differ and how. Adding group-level moderators to the structural relations may still fail to explain the differences (e.g., Brandt et al., 2021).

In scenarios with many groups, some groups likely have equivalent structural relations—for example, due to a shared cultural background—so that “latent classes” or “clusters” of groups with common relations arise. Identifying these clusters would thus be an intuitive and efficient alternative to pairwise comparisons, which can be done by means of mixture modeling (McLachlan and Peel, 2000).

Before clustering groups on structural relations, we need to consider whether these relations can be validly compared across the groups. In social sciences, the constructs of interest are typically “latent” variables that are measured indirectly through indicator variables, like questionnaire items, which contain measurement error. In SEM, the latent nature of the constructs—also called “factors”—is accounted for by estimating a measurement model (MM) for each construct (capturing how it is measured by observed indicators), as well as the relations between the constructs—which are part of the structural model (SM). For ensuring comparability of constructs across groups, some degree of “measurement invariance” (MI) should hold, meaning that the constructs are measured equivalently across groups so that differences in the (relations between) constructs are not due to differences in how they are measured.

Different levels of MI pertain to different subsets of measurement model parameters. Configural invariance concerns equivalence of the factor loading structure (i.e., the number of factors and the pattern of zero and non-zero loadings) across groups, where factor loadings capture the factor-indicator relations so that configural invariance implies that a construct is measured by the same set of items in all groups. Metric invariance concerns equality of the factor loadings (i.e., the strength and direction of the factor-indicator relations). Scalar invariance requires equality of item intercepts. Lastly, residual invariance implies equality of the items' residual or “unique” variances (i.e., not explained by the factor). To make accurate between-group comparisons of construct relations, metric invariance (Davidov et al., 2012) should hold across groups. With many groups, however, measurement non-invariance is often encountered (e.g., Boer et al., 2018; Rutkowski and Svetina, 2014). If equality does not hold for all factor loadings, partial metric invariance still enables valid comparisons of structural relations, meaning that some loadings are invariant while others differ (Byrne et al., 1989; Pokropek et al., 2019). Non-invariance of item intercepts and unique variances do not invalidate the comparison of structural relations. To avoid incorrect (biased) estimates of structural relations, differences in loadings, intercepts and unique variances must be accounted for within the SEM model (Chen, 2008; Guenole and Brown, 2014; Pokropek et al., 2019).

From this reflection on MI, we conclude that differences in structural relations are not the only between-group differences we can encounter in SEM, but they are the only differences we want to capture by clustering. However, traditional mixture SEM methods (Arminger and Stein, 1997; Dolan and van der Maas, 1998; Jedidi et al., 1997) capture differences in all SEM parameters with clustering—including MM parameters (loadings, intercepts, and unique variances) as well as SM parameters (structural relations and factor means)—except for parameters that are constrained to be equal across clusters. As a result, this method may need many clusters to capture both measurement non-invariances and structural differences, or it may mix up the two sources of differences or capture the most dominant differences only, failing to isolate differences in structural relations.

To solve this shortcoming, Perez Alonso and colleagues (Perez Alonso et al., 2024) proposed Mixture Multigroup SEM (MixMG-SEM), which uses mixture modeling to cluster groups based on their structural relations while accounting for potential measurement non-invariance by keeping the MM partially group-specific. Unlike other mixture SEM approaches, MixMG-SEM ensures that the clustering is solely based on structural relations rather than (also) on differences in how the constructs are measured. However, when the sample size per group is small (say 50 or less, or barely higher than the number of variables), taking a multigroup approach to capturing measurement non-invariance may not converge or result in less accurate and inefficient estimates of the group-specific measurement parameters, which may propagate into the SM and affect the estimates of the structural relations as well as the clustering based on these relations. Therefore, in this paper, we propose a more parsimonious alternative: Mixture Multilevel SEM (MixML-SEM). It uses the multilevel approach to capturing measurement non-invariance, while comparing structural relations across groups by means of a mixture clustering. Hence, it accounts for between-group differences in measurement parameters by means of random effects at the group-level, reducing the number of parameters and thus resulting in more efficient estimates (Hox et al., 2017). Note that at least 30 or 50 groups are needed to obtain valid estimates of the random effects (Leitgöb et al., 2023), however.

While the multilevel approach is commonly favored for modeling differences among many groups, in MixML-SEM, we employ it specifically for the MM but not for the structural relations. This distinguishes MixML-SEM from ML-SEM. As mentioned above, modeling variance in structural relations does not help in pinpointing similarities and differences across groups. Furthermore, group-specific estimates derived from random effects are systematically biased toward the overall mean parameter value, especially when the sample size per group are small (Hox et al., 2017), impairing the comparisons of group-specific regression coefficients resulting from ML-SEM. In contrast, MixML-SEM provides more accurate estimates of the structural relations by combining information from multiple groups within a cluster when estimating the regression coefficients (i.e., they are directly estimated as cluster-specific parameters), which alleviates the effect of having small sample size per group (as long as the clusters are still sufficiently separated).

For estimating MixML-SEM, we build on the “Structural-After-Measurement” (SAM) framework presented by Rosseel and Loh (2024), which decouples the estimation of the measurement and structural parts of the SEM model. For the estimation of MixML-SEM, we adopt a tailored variant of the local SAM approach, where the estimation of structural relations operates directly on the covariances between factors and, thus, no longer involves the measurement parameters. Specifically, a multilevel factor analysis is performed per factor, while accounting for potential measurement non-invariances with random effects. Factor scores (i.e., estimated latent variable scores) are extracted for each factor. Then, these factor scores are used as scores on a single indicator for the factor, for which measurement parameters are derived, and Croon's correction (Croon, 2002) is applied to compute the factor covariances. These factor covariances are the input for the final step, which boils down to a mixture multigroup path model, estimating the mixture clustering of the groups and the cluster-specific regression relations among the factors.

To conclude, this paper presents MixML-SEM for efficiently comparing structural relations across many groups (by means of mixture modeling), while handling measurement non-invariances with a low number of parameters (by means of multilevel modeling). The paper is organized as follows: Firstly, we provide a comprehensive description of the specification and estimation of MixML-SEM. Next, we present simulation studies assessing the performance of MixML-SEM in terms of model estimation and model selection, comparing it to ML-SEM. We then demonstrate the empirical value of MixML-SEM using data on how the perceived social pressure to be happy relates to people's life satisfaction. Finally, we summarize the main findings and discuss limitations of the study as well as directions for future research.

## Specification and estimation of MixML-SEM

In Step 1 of MixML-SEM, the measurement model (MM) is estimated by performing a Multilevel Confirmatory Factor Analysis (ML-CFA) per factor (i.e., per construct). In Step 2, the factor scores obtained in Step 1 are used as scores on a single indicator (with fixed measurement parameters) for each respective factor and Croon's correction (Croon, 2002) is applied to obtain bias-corrected factor covariances. In Step 3, the structural model (SM)—including the clustering of the groups and the cluster-specific structural relations—is estimated using an Expectation-Maximization (Dempster et al., 1977) algorithm. Below, we elaborate on each step, including the relevant model specifications. Then, we discuss how to determine an essential aspect of the model specification: the number of clusters.

### Step 1: ML-CFA with measurement non-invariances

The MM captures how the indicators (observed variables) relate to the constructs of interest (latent variables). For each construct (factor) *q* (*q* = 1, …, *Q*), **x**_*n*_*g*__ denotes the observed scores on the *J*_*q*_ indicator items for individual *n* nested within group *g* (*g* = 1, …, *G*), which are modeled as follows:


(1) $$\begin{array}{l} {\text{x}}_{n_{g}}=\tau_{g}+\lambda_{g}\eta_{n_{g}}+\epsilon_{n_{g}}\;with\;\epsilon_{n_{g}}\sim \;MVN\left(\text{0},\Theta_{g}\right) \end{array}$$

where **τ**_*g*_ is a *J*_*q*_-dimensional vector of intercepts for group *g*, **λ**_*g*_ denotes a *J*_*q*_-dimensional vector of factor loadings for group *g*, quantifying the expected change in the item scores due to a one-unit change in the latent variable score η_*n*_*g*__, and **ϵ**_*n*_*g*__ is a *J*_*q*_-dimensional vector of residuals, where the diagonal of **Θ**_*g*_ contains the items' unique variances in group *g*, representing variance that is unexplained by the underlying construct. To set the scale of the latent variables, we adopt the marker variable approach, where one loading per factor is fixed to one for each group, so that a one-unit change of a factor has the same meaning in all groups.

Note that we formulated the MM for each factor separately, in line with the “measurement blocks” concept introduced by Rosseel and Loh (2024) in their SAM approach. They recommend estimating the MM of each latent variable separately, which corresponds to having one factor within each measurement block. This strategy streamlines computational efficiency by avoiding estimating one larger model with more parameters and it enhances the model's robustness against potential misspecifications, such as unmodeled cross-loadings.

Multigroup confirmatory factor analysis (MG-CFA; Meredith and Teresi, 2006; Sörbom, 1974) allows fitting MMs for multiple groups and determining which parameters are invariant. The invariance of a subset of measurement parameters holds when imposing their equality across groups (e.g., **λ**_*g*_**=λ** for *g* = 1, …, *G*) does not significantly worsen the fit of the model. In MG-CFA, a non-invariant measurement parameter is estimated separately for each group, which results in a large number of parameters to be estimated. In multilevel terminology, this is referred to as the “fixed effect approach,” which is known to result in less efficient parameter estimates, meaning that the group-specific parameters are estimated with greater uncertainty, especially for small groups. Small-sample bias may also make the group-specific parameter estimates less accurate. Therefore, ML-CFA has gained prominence as a parsimonious alternative for capturing heterogeneity in parameters across many groups (Kim et al., 2016; Muthén, 1991, 1994), a trend supported by the availability of statistical software like Mplus (Muthén and Muthén, 1998). Instead of estimating separate measurement parameters for each group, ML-CFA estimates a single MM for all groups, allowing measurement parameters to vary randomly across groups. This variation is modeled through random effects, which essentially impose a certain distribution on the variation in parameters. For these random effects, only the mean and variance are estimated as parameters, which results in more efficient estimation (Hox et al., 2017). As mentioned in the Introduction, group-specific estimates can be derived from the random effects; however, these estimates are subject to shrinkage bias toward the overall mean.

When estimating MixML-SEM, we start by performing a separate ML-CFA for each factor using the Bayes estimator (Asparouhov and Muthén, 2012). Note that, since our primary focus is the comparison of structural relations and not of latent means, the mean structure of the data (i.e., the group-specific means) is removed by centering the observed scores per item within each group (therefore, **τ**_*g*_ = **0** for *g* = 1, …, *G*). This also lowers computational demands. For individual *n* in group *g*, the ML-CFA model for a single factor with random loadings is then expressed as follows:

Level-1 Model:


(2) $$\begin{array}{l} {\text{x}}_{n_{g}}=\lambda_{g}\eta_{n_{g}}+\epsilon_{n_{g}}\;with\;\epsilon_{n_{g}}\sim \;MVN\left(\text{0},\Theta_{g}\right) \end{array}$$

Level-2 Model:


(3) $$\begin{array}{lll} \lambda_{jg} & = & \gamma_{\lambda_{j}}+u_{\lambda_{jg}}\;with\;u_{\lambda_{jg}}\sim N\left(0,\;\sigma_{\lambda_{j}}\right) \end{array}$$

(4) $$\begin{array}{lll} \theta_{jg} & = & \gamma_{\theta_{j}}+u_{\theta_{jg}}\;with\;log\left(\theta_{jg}\right)\sim N\left(\gamma_{\theta_{j}},\;\sigma_{\theta_{j}}\right) \end{array}$$

(5) $$\begin{array}{lll} \phi_{qg} & = & \gamma_{\phi_{q}}+u_{\phi_{qg}}\;with\;log\left(\phi_{qg}\right)\sim N\left(\gamma_{\phi_{q}},\;\sigma_{\phi_{q}}\right) \end{array}$$

At Level-1, **λ**_*g*_ refers to the *J*_*q*_-dimensional vector of factor loadings for group *g*, η_*n*_*g*__ is the latent variable score and **ϵ**_*n*_*g*__ the within-level error term for individual *n*_*g*_. At Level-2, random effects are included for each non-invariant parameter (i.e., for each random loading λ_*jg*_, random unique variance θ_*jg*_, and random factor variance ϕ_*qg*_). Random effects for loadings are also referred to as “random slopes.” Random loadings λ_*jg*_ are assumed to be normally distributed. In Equation 3, γ_λ_*j*__ refers to the average slope, and *u*_λ__*j*__*g*___ is the group-specific deviation from the average slope. While only the mean (γ_λ_*j*__) and the variance (σ_λ_*j*__) of the random slopes are estimated as parameters, it is possible to obtain group-specific loading estimates from the posterior distributions when using the Bayes estimator (i.e., posterior mean estimates, e.g., Asparouhov and Muthén, 2012). For an invariant loading, *u*_λ_*jg*__ becomes 0 and λ_*jg*_**=**λ_*j*_ for all groups. As highlighted in the Introduction, at least partial metric invariance should hold for the between-group comparisons of structural relations to be valid. This implies that at least some loadings should be invariant across the groups, so that the vector of loadings contains both invariant and non-invariant ones. If full metric invariance holds, no random slopes are needed and the vector of loadings is fully equal across groups, so that **λ**_*g*_ = **λ** for *g* = 1, …, *G*.

Differences in unique variances should also be captured by random effects (Equation 4), so that **Θ**_*g*_ can also contain a combination of invariant and non-invariant unique variances—depending on the results of the MI testing. Additionally, it is reasonable to expect differences in factor variances across groups. Thus, one should also specify random factor variances, ϕ_*qg*_, when necessary (Equation 5). Since it is not suitable to assume random variances (i.e., unique variances θ_*jg*_ and factor variances ϕ_*qg*_) following a normal distribution, the log of the variance is modeled by a normal distribution (e.g., “logv,” see Muthén and Asparouhov, 2023). Throughout the paper, we assumed the factor loadings and unique variances to be partially group-specific and factor variances to be fully group-specific, so we always refer to them with a subscript *g*.

As mentioned above, it is necessary to determine beforehand which measurement parameters should be specified as non-invariant (i.e., with random effects) and which ones as invariant (i.e., without random effects). Hence, MI testing should precede MixML-SEM. Even though MG-CFA is the most commonly used method, the MI test can also be performed with ML-CFA (Kim et al., 2017; Leitgöb et al., 2023). To evaluate MI, one can use the random effects directly. To assess whether (partial) metric invariance holds, the factor loadings are specified as random across groups (as in Equation 3) and, then, one can test—for each loading—whether the variance of the random loadings σ_λ_*j*__ is non-zero (Asparouhov and Muthén, 2012; Leitgöb et al., 2023), which implies that the corresponding loading is non-invariant. For unique variances and factor variances, one can also test whether the variance of their random effect is non-zero. Note that the MI testing method from Jak et al. (2013) is not applicable in our context, as we removed the mean structure and thus the between-level (co)variances.

Once the non-invariant parameters are identified and accounted for by random effects, we obtain the ML-CFA model that corresponds to the first step of MixML-SEM. Throughout this paper, Step 1 of MixML-SEM is performed by means of Mplus and the R-package MplusAutomation (Hallquist and Wiley, 2018), using the Bayes estimator with default, non-informative priors. Upon estimating the MM for each factor, the posterior distributions (i.e., posterior means and standard deviations) of the factor scores (i.e., estimated latent variable scores) are appended to the data file. These values are used in Step 2. For more details, see Supplementary material S1.

### Step 2: single-indicator approach to obtain group-specific factor covariances

The goal of Step 2 is to obtain group-specific factor covariances, denoted as $\Phi_{g}^{s2}$. Factor scores are estimates of the true latent variable scores that contain error, so when they are used in regression or path analysis as if they were the true latent variable scores, the regression estimates may be biased (Devlieger and Rosseel, 2017, 2020). Croon developed a method to correct for the bias (Croon, 2002). In a multigroup setting, Croon's formula describes the relation between the factor score covariances (*cov*(**F**_*g*_)) and the true latent variable covariances (*cov*(**η**_*g*_)) as follows:


(6) $$cov\left(F_{g}\right)=\;A_{g}\Lambda_{g}cov\left(\eta_{g}\right)\Lambda_{g}^{\prime }A_{g}^{'}+A_{g}\Theta_{g}A_{g}^{'}$$

where **F**_*g*_ is the matrix containing the factor scores for all individuals of group *g*, **A**_*g*_ is the group-specific factor score matrix, containing the coefficients needed to convert the item scores into factor scores, **Λ**_*g*_ is the factor loading matrix for group *g*, and **Θ**_*g*_ is the unique variance matrix for group *g*.

As mentioned in the Introduction, random effect estimates for the group-specific factor loadings in **Λ**_*g*_ and unique variances in **Θ**_*g*_ can be biased, especially when the within-group sample size is small. Therefore, we opt to use only the estimated factor scores from Step 1, which contain all necessary information to proceed (Vermunt, 2024). The factor scores can serve as a single “observed” indicator of the factor, which reduces the data's dimensionality. We can derive the measurement parameters of these single indicators from the estimated factor scores and their standard deviations (see below). The single-indicator approach is similar to the factor score regression approach with Croon's correction (Croon, 2002; Vermunt, 2024), with the difference that we now no longer use the measurement parameters of the observed indicators to perform the correction in Equation 6. To make sure that the mean of the estimated factor scores is exactly zero per group, we centered them per group.

The group-specific loading for factor *q* in group *g*, denoted as λ_*qg*_, is equal to the reliability of the factor scores. The reliability is defined as the ratio of the variance of the factor scores within group *g* (i.e., the variance explained by the items) to the group-specific factor variance (i.e., total variance of the factor):


(7) $$\begin{array}{l} \lambda_{qg}=\frac{var\left(E\left(f_{q_{n_{g}}}\right)\right)}{\phi_{qg}} \end{array}$$

In the Bayesian framework, the factor score for each individual *n*_*g*_ is considered a random variable with a distribution. For factor *q*, *E*(*f*_*q*__*n*__*g*___) represents the posterior mean of this distribution for individual *n*_*g*_ (i.e., the mean of the posterior distribution)—and corresponds to the estimated factor score—and *var*(*E*(*f*_*q*__*n*__*g*___)) stands for the variance of the estimated factor scores across all individuals *n* within group *g*. The group-specific unique variance for factor *q* is set to ϕ_*qg*_λ_*qg*_(1−λ_*qg*_). The group-specific factor variance ϕ_*qg*_ can be obtained using the posterior means and variances of the factor scores as follows:


(8) $$\begin{array}{l} \phi_{qg}=var\left(E\left(f_{q_{n_{g}}}\right)\right)+\;E\left(var\left(f_{q_{n_{g}}}\right)\right) \end{array}$$

where *var*(*f*_*q*__*n*__*g*___) represents the variance of this distribution (i.e., the square of the estimated standard deviation obtained from Step 1) for individual *n*_*g*_ and *E*(*var*(*f*_*q*__*n*__*g*___)) represents the mean of the variance across all individuals *n* within group *g*. Technically, the group-specific factor variance ϕ_*qg*_ can also be obtained directly from the random effects of the factor variances in Step 1, but when using these random effects estimates in Equation 7, λ_*qg*_ can become larger than one, leading to a negative unique variance. Therefore, we use Equation 8 to derive group-specific factor variances, ϕ_*qg*_.

By gathering these parameters for all factors, we obtain the *Q*×*Q* group-specific factor loadings $\hat{\Lambda_{g}}$ and *Q*×*Q* group-specific unique variances $\hat{\Theta_{g}}$ for the factor scores as single indicators. Note that $\hat{\Lambda_{g}}$ and $\hat{\Theta_{g}}$ are equivalent to **A**_*g*_**Λ**_*g*_ and **A**_*g*_**Θ**_*g*_**A**_*g*_ in Equation 6, respectively (Vermunt, 2024). The group-specific factor covariance matrices $\Phi_{g}^{s2}=cov\left(\eta_{g}\right)$, which serve as the input for Step 3, can thus be derived as follows:


(9) $$\begin{array}{l} \Phi_{g}^{s2}=\;\hat{\Lambda_{g}^{-1}}(cov(F_{g})-\hat{\Theta_{g}}){\left(\hat{\Lambda_{g}^{\prime }}\right)}^{-1} \end{array}$$

Note that, instead of these factor covariances, it is theoretically possible to use the factor scores themselves as the input for Step 3, with measurement parameters that are fixed to $\hat{\Lambda_{g}}$ and $\hat{\Theta_{g}}$. This would be a global SAM version of Step 3, which is computationally slow despite the dimension reduction due to the single-indicator approach. Therefore, we apply this intermediate Step 2 to obtain group-specific factor covariance matrices so that we no longer need to work with the measurement parameters in Step 3 (i.e., the local SAM approach).

### Step 3: structural model with mixture clustering of the groups

This step corresponds to the second step of the MixMG-SEM method introduced by Perez Alonso et al. (2024). It aims to find the underlying clusters of groups and their cluster-specific structural relations. Thus, the SM is conditional on the cluster membership *z*_*gk*_, which indicates whether group *g* belongs to cluster *k*:


(10) $$\begin{array}{l} \left[\eta_{n_{g}}|z_{gk}=1\right]={\text{B}}_{k}\eta_{n_{g}}+\zeta_{n_{g}} \end{array}$$

where **B**_*k*_ contains the cluster-specific regression coefficients between latent variables, and **ζ**_*n*_*g*__ indicates the disturbances of these regressions. Under the assumption *E*(**ζ**_*n*_*g*__)**=0** and *cov*(**ζ**_*n*_*g*__)**=Ψ**_*g*_, the model-implied factor covariance matrix is computed as:


(11) $$\begin{array}{l} \Phi_{gk}={\left(\text{I}-{\text{B}}_{k}\right)}^{-1}{\text{Ψ}}_{gk}{\left(\text{I}-{\text{B}}_{k}\right)}^{-1^{\prime }} \end{array}$$

Note that the residual factor covariances **Ψ**_*gk*_ are specified as both group- and cluster-specific as they depend on the cluster-specific regression coefficients **B**_*k*_ but should not affect the cluster memberships. Estimating the SM involves minimizing the discrepancy between the group-specific factor covariance matrices obtained in Step 2, $\Phi_{g}^{s2}$, and their corresponding model-implied reconstructions, **Φ**_*gk*_. Note that the latter will differ from the former when the SM is not saturated.

MixML-SEM assumes that latent variable scores **η**_*n*_*g*__ are sampled from a mixture of *K* multivariate normal distributions where all latent variable scores of a group (gathered in **H**_*g*_) are assumed to be sampled from the same distribution. More specifically, the MixML-SEM for group *g* is written as follows:


(12) $$\begin{array}{l} f\left({\text{H}}_{g};\upsilon \right)=\overset{K}{\underset{k=1}{\sum }}\pi_{k}\;\overset{N_{g}}{\underset{n_{g}=1}{\prod }}MVN\left(\eta_{n_{g}};\alpha_{g}{,\Phi }_{gk}\right) \end{array}$$

Here, *f* represents the total population density function, υ is the set of population parameters. π_*k*_ stands for the prior probability of a group *g* belonging to cluster *k* (with $\overset{K}{\underset{k=1}{\sum }}\pi_{k}$ = 1). The mean vector **α**_*g*_ is **0** due to centering and covariance matrix **Φ**_*gk*_ is decomposed as in Equation 11.

The unknown parameters υ are estimated by maximizing the following log-likelihood function:


(13) $$\begin{array}{l} \log L_{\eta }=\log\left(\prod_{g=1}^{G}\sum_{k=1}^{K}\pi_{k}\frac{1}{{\left(2\pi \right)}^{\frac{Q}{2}}|(\Phi_{gk}){|}^{\frac{1}{2}}}\exp{\left(-\frac{1}{2}tr\left(\Phi_{g}^{s2}\Phi_{gk}^{-1}\right)\right)}^{N_{g}}\right)\\ \;=\sum_{g=1}^{G}\log\left(\sum_{k=1}^{K}\pi_{k}\frac{1}{{\left(2\pi \right)}^{\frac{Q}{2}}|(\Phi_{gk}){|}^{\frac{1}{2}}}\exp{\left(-\frac{1}{2}tr\left(\Phi_{g}^{s2}\Phi_{gk}^{-1}\right)\right)}^{N_{g}}\right) \end{array}$$

where $\Phi_{g}^{s2}$ is the group-specific factor covariance matrix from Step 2 (Equation 9), and **Φ**_*gk*_ is the group- and cluster-specific factor covariance matrix from Step 3 (Equation 11). We use an Expectation-Maximization (EM; Dempster et al., 1977) algorithm to optimize this log-likelihood function. In the E-step, the algorithm estimates the expected values of the cluster memberships of the groups given the current parameter estimates; that is, the classification probabilities ${\hat{z}}_{gk}$. In the M-step, the algorithm maximizes the unknown parameters υ given the expected cluster memberships from the E-step by calling lavaan (Rosseel, 2012). Note that the M-step includes a bias correction procedure to get **Ψ**_*gk*_. Readers can consult Perez Alonso and colleagues' paper (Perez Alonso et al., 2024), Appendix A, for a deeper dive into the technical details of Step 3. The E- and M-steps are iterated until convergence is reached, which is when the change in log-likelihood between iterations becomes sufficiently small (e.g., <1 × 10^−6^). A multi-start procedure, starting from multiple random partitions, is used to avoid convergence to local maxima. The solution with the highest log-likelihood is selected as the final result.

### Model selection

Because the number of clusters underlying the data is unknown in real life, we compare models with different numbers of clusters using the following methods: Bayesian Information Criterion (BIC; Schwarz, 1978), Akaike Information Criterion (AIC; Akaike, 1974), and the convex hull procedure (CHull; Ceulemans and Kiers, 2006). BIC combines the model's log-likelihood with a penalty based on the number of parameters:


(14) $$\begin{array}{l} BIC=-2logL+P\log\left(SS\right) \end{array}$$

Here, *P* is the number of free parameters and *SS* is the sample size. The model with the smallest BIC value is selected. For MixML-SEM, *P* is the sum of the number of mixing proportions (minus one restriction), the number of cluster-specific regression coefficients, the number of group- and cluster-specific factor (co-)variances (counting only one set per group, since the model assumes each group to belong to one cluster only), and the number of measurement parameters. Recall that, from Step 2 onwards, the factor scores are used as single indicators for the latent variables. Therefore, we include the number of loadings $\hat{\Lambda_{g}}$ and unique variances $\hat{\Theta_{g}}$ for the factor scores as the number of measurement parameters in *P*. In simulation studies involving the mixture multigroup approach (De Roover, 2021; De Roover et al., 2022; Perez Alonso et al., 2025), it was found that the BIC performed better when *SS* is equal to the number of groups *G* (BIC_*G*_) rather than the total number of observations *N* (BIC_*N*_), which is why we focus on BIC_*G*_ throughout the paper.

In case of small sample sizes and low cluster separation, AIC was found to outperform BIC for some related methods (De Roover et al., 2022; Kim et al., 2017), but not all (De Roover, 2021). AIC penalizes model complexity as follows:


(15) $$\begin{array}{l} AIC=-2logL+2P \end{array}$$

Moreover, the CHull has been shown to be a valuable alternative to BIC and AIC (Bulteel et al., 2013; De Roover, 2021; De Roover et al., 2022). It balances the logL and the number of free parameters by means of a generalized scree test, selecting the model with the highest scree ratio. Note that a limitation of CHull is that it always selects at least two clusters, because the scree ratio cannot be computed for a one-cluster solution, but visual inspection of the CHull plot can help identify whether a clear elbow is present. If not, an underlying clustering is less likely.

Since we use the estimated factor scores as the single “observed” indicator in Steps 2 and 3, we use the following loglikelihood in BIC, AIC, and CHull:


(16) $$\begin{array}{l} \log L=\log(\prod_{g=1}^{G}\sum_{k=1}^{K}\pi_{k}\prod_{n_{g}=1}^{N_{g}}\frac{1}{{\left(2\pi \right)}^{\frac{Q}{2}}|(\hat{\Sigma_{gk}}\;){|}^{\frac{1}{2}}}\\ \;\exp\left(-\frac{1}{2}(f_{n_{g}}-{\bar{f}}_{g}{)}^{\prime }{\hat{\Sigma_{gk}}}^{-1}(f_{n_{g}}-{\bar{f}}_{g}))\right)\\ \;=\sum_{g=1}^{G}\log(\sum_{k=1}^{K}\pi_{k}\prod_{n_{g}=1}^{N_{g}}\frac{1}{{\left(2\pi \right)}^{\frac{Q}{2}}|(\hat{\Sigma_{gk}}\;){|}^{\frac{1}{2}}}\\ \;\exp\left(-\frac{1}{2}(f_{n_{g}}-{\bar{f}}_{g}{)}^{\prime }{\hat{\Sigma_{gk}}}^{-1}(f_{n_{g}}-{\bar{f}}_{g}))\right) \end{array}$$

where **f**_*n*_*g*__ refers to the *Q*-dimensional vector of estimated factor scores of individual *n*_*g*_, ${\bar{f}}_{\text{g}}$ refers to the mean of the estimated factor scores for each group, which equals zero due to centering, $\hat{\Sigma_{gk}}$ refers to the model-implied covariance matrix of the *Q* single indicators, which is equal to:


(17) $$\begin{array}{l} \hat{\Sigma_{gk}}=\hat{\Lambda_{g}}\Phi_{gk}\hat{\Lambda_{g}}+\hat{\Theta_{g}}\;={\hat{\Lambda_{g}}\;\left(\text{I}-{\text{B}}_{k}\right)}^{-1}{\text{Ψ}}_{gk}{\left(\text{I}-{\text{B}}_{k}\right)}^{-1^{\prime }}\hat{\Lambda_{g}}+\hat{\Theta_{g}} \end{array}$$

Note that using the loglikelihood for the observed items would be too complicated since obtaining a valid loglikelihood requires integrating out the random effects for the non-invariant measurement parameters and the factor variances (see Step 1).

## Simulation studies

In Simulation Study 1, we evaluated the performance of MixML-SEM when the number of clusters is assumed to be known, and compared it to ML-SEM. Then, in Simulation Study 2, we investigated whether the correct number of clusters is selected for MixML-SEM by BIC_*G*_, AIC, and/or CHull.

### Simulation study 1

The goal of the Simulation Study 1 was two-fold: Firstly, we aimed to evaluate the performance of MixML-SEM in terms of parameter and cluster recovery when the number of clusters is known. Secondly, we compared it to ML-SEM, where group-specific regression coefficients were derived from random effects. Specifically, we performed ML-SEM with Mplus and the R-package MplusAutomation (Hallquist and Wiley, 2018), where the SM and MM were estimated simultaneously with random effects for capturing differences. The following factors were manipulated:

1. Total number of groups *G* (2 levels): 48, 96;
2. Number of clusters *K* (2 levels): 2, 4;
3. Small groups *N*_*g*_ (2 levels): 25, 50;
4. Small groups proportion (5 levels): 0, 0.25, 0.5, 0.75, 1;
5. Large groups *N*_*g*_ (2 levels): 100, 200;
6. Size of regression parameters β (3 levels): 0.2, 0.3, 0.4;
7. Reliability (2 levels): high, low;
8. Within-group samples: fixed, random.

We included two levels of the total number of groups *G*, with a minimum of 48 groups, based on the recommendation that at least 30 or 50 groups are needed to obtain valid estimates of random effects (Leitgöb et al., 2023). Because more groups imply more information on cluster-specific regression estimates (i.e., a larger within-cluster sample size), we hypothesize that the performance of MixML-SEM will improve with a higher number of groups.

We considered two levels of the number of clusters *K* underlying the groups: two or four. A higher number of clusters lowers the within-cluster sample size and is thus expected to lower the performance of MixML-SEM. Additionally, it raises the complexity of determining the cluster memberships (i.e., more posterior classification probabilities) for each group, making the recovery of clusters more intricate. Here, we focused on balanced cluster sizes, where all clusters contained an equal number of groups. In practice, cluster recovery is likely to be more challenging when cluster sizes are unbalanced, as was demonstrated by Perez Alonso et al. (2024).

As mentioned in the Introduction, an advantage of MixML-SEM is combining information from multiple groups within a cluster when estimating the (cluster-specific) regression coefficients. In this way, the regression estimates for small groups benefit from the presence of large groups within the same cluster, if any. If only small groups are combined in a cluster and their cluster memberships are very uncertain (i.e., classification probabilities <1), this may affect the estimation of the cluster-specific regression estimates. Therefore, we generated data with a mix of small and large groups, which is also a realistic setting. To this end, we initially randomly selected a specific number of groups per cluster which were assigned a small *N*_*g*_ of either 25 or 50, where this number of groups was determined by the small groups proportion of 0, 0.25, 0.5, 0.75, or 1. Note that this proportion is applied to each cluster, so that the equality of the within-cluster sample sizes is preserved. Subsequently, the other groups were assigned a large *N*_*g*_ of either 100 or 200. A larger proportion of small groups lowers the within-cluster sample size, and is thus expected to lower the performance. To summarize, the group sizes are determined by three factors: the large *N*_*g*_, the small *N*_*g*_, and the small groups proportion. Note that the within-cluster sample sizes are determined by all the abovementioned factors.

**Figure 1:**
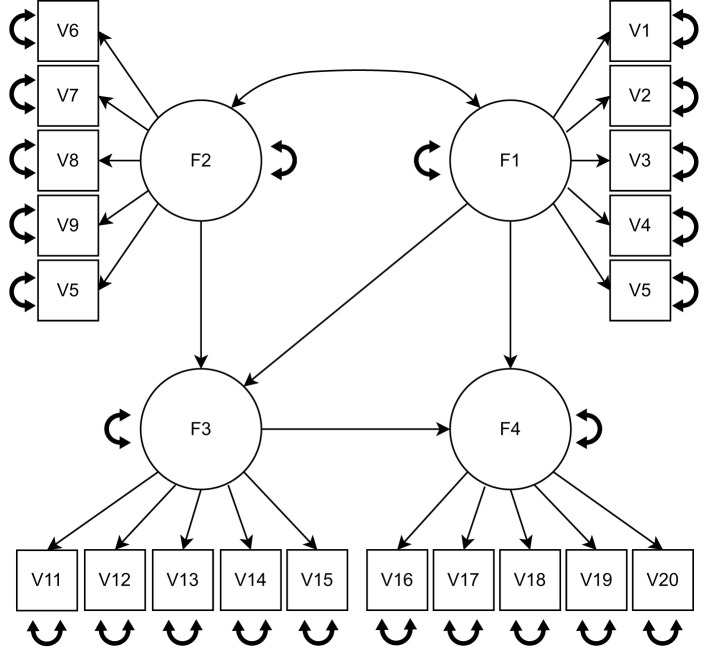
The model used for the data generation. F1 and F2 are exogenous variables, F3 and F4 are endogenous variables.

**Figure 2:**
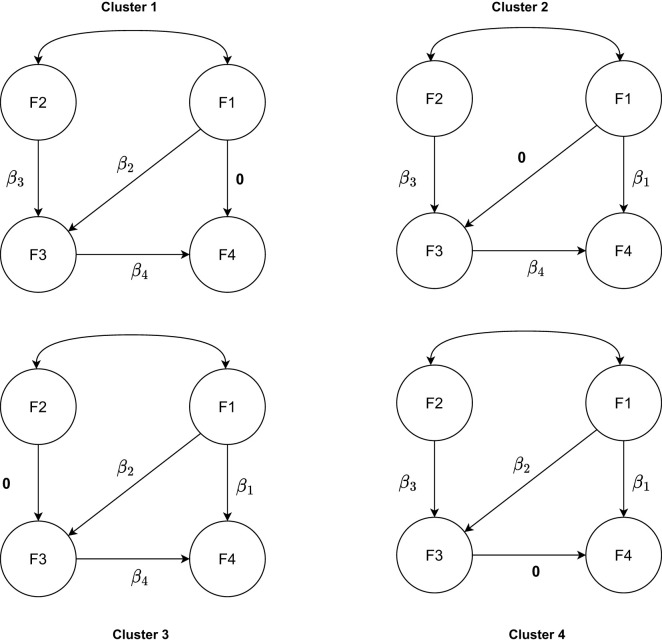
The regression parameters between the latent variables depending on the cluster.

The data were generated by a SEM model with four latent variables, each measured by five items (see Figure 1), as was also used by Perez Alonso et al. (2024). As mentioned above, we assume the latent variable scores for group *g* follow a multivariate normal distribution with covariance matrix **Σ**_*gk*_**, ** which is determined by the parameters: **B**_*k*_, **Ψ**_*gk*_, **Λ**_*g*_ and **Θ**_*g*_. We first defined the cluster-specific regression parameters **B**_*k*_. As illustrated in Figure 2, for each cluster, one of them was set to zero, while the other regression parameters were set equal to the size of regression parameters β. Therefore, for each pair of clusters, the difference between them pertains to two regression coefficients and the size of each difference is equal to β. We considered three levels of regression parameters. The larger the size of regression parameters β, the more separated the clusters become and the easier the cluster recovery will be.

Secondly, we generated the group- and cluster-specific residual factor covariances **Ψ**_*gk*_. For the exogenous variables *F*1 and *F*2, we sampled the group-specific covariances (*Cov*(*F*1, *F*2)_*g*_) from a Wishart distribution, with variances (*Var*(*F*1)_*g*_, *Var*(*F*2)_*g*_) varying around 1 and their covariance varying around 0. Across groups within simulated data sets, *Var*(*F*1) and *Var*(*F*2) varied from 0.608 to 1.389 (mean = 0.952, *SD* = 0.166), whereas *Cov*(*F*1, *F*2) varied across groups from −0.279 to 0.279 (mean = 0.000, *SD* = 0.117). For the endogenous variables *F*3 and *F*4, the total variances (*Var*(*F*3)_*g*_, *Var*(*F*4)_*g*_) were sampled separately from a log-normal distribution (with the mean on the log scale set to 0). Their residual variances depended on the regression parameters. For *F*3, it was $Var{\left(F3\right)}_{g}-\left(\beta_{2,k}^{2}Var{\left(F1\right)}_{g}+\beta_{3,k}^{2}Var{\left(F2\right)}_{g}+2\beta_{2,k}\beta_{3,k}{Cov\left(F1,F2\right)}_{g}\right)$. For *F*4, it was$Var{\left(F4\right)}_{g}-\left(\beta_{1,k}^{2}Var{\left(F1\right)}_{g}+\beta_{4,k}^{2}Var{\left(F3\right)}_{g}+2\beta_{1,k}\beta_{4,k}\left(\beta_{2,k}Var{\left(F1\right)}_{g}+\beta_{3,k}Cov{\left(F1,F2\right)}_{g}\;\right)\right)$.

Thirdly, we specified the group-specific loading matrices **Λ**_*g*_ and unique variances **Θ**_*g*_, based on the reliability. The first loading of each latent variable was fixed to 1 to set the scale of the latent variable. Per latent variable, the loadings and unique variances of the second and third indicator were set to be non-invariant. When the reliability level was high, the invariant loadings were set to $\sqrt{0.6}$ and their unique variances to 0.4; when the reliability was low, the invariant loadings were set to $\sqrt{0.4}$ and their unique variances to 0.6. Meanwhile, the non-invariant loadings were sampled from a normal distribution with a mean of either $\sqrt{0.6}$ or $\sqrt{0.4}$ and a variance of 0.1 for all groups. The non-invariant unique variances were sampled from a log-normal distribution (with the standard deviation on the log scale set to 0.25 and the mean to −0.948 or −0.542 to generate unique variances around 0.4 and 0.6, respectively, for the high and low reliability conditions). When the reliability is higher, we expect a better recovery of the MM in MixML-SEM, potentially leading to a better cluster recovery.

Finally, after defining all the necessary parameters, data were sampled from a multivariate normal distribution *MVN***(0, Σ**_*gk*_**)** for each group, either with fixed or random within-group samples. This was operationalized using the “empirical” argument in the mvrnorm function from the MASS package (Venables and Ripley, 2002). With empirical = TRUE, the covariance matrix of the sampled data exactly matches the specified **Σ**_*gk*_. This setting corresponds to the empirical situation where all individuals nested within groups are included in the sample (e.g., including all pupils of a classroom), or when only the specific set of individuals in the sample is of interest, without intending to draw conclusions about the broader population of individuals within a group. In contrast, with empirical = FALSE, the within-group samples are regarded as a random sample from a larger population within a certain group (e.g., inhabitants of a country) and one intends to draw conclusions about that entire population. In the latter case, the sample's covariance matrix will differ from **Σ***_gk_* due to sampling fluctuations, and more so for smaller group sizes. Thus, we expect the recovery of the clusters and parameters to be more challenging in the random conditions, especially when (more) groups are smaller.

We generated 50 replications per cell of the design, yielding 48,000 data sets in total, using R version 4.4 (R Core Team, 2022). All data sets were analyzed using MixML-SEM with 50 random starts, and ML-SEM. For both methods, the measurement non-invariances were correctly specified as group-specific parameters. The analyses were performed on a supercomputer consisting of 2 Intel Xeon Platinum 8468 CPUs (Sapphire Rapids). The average computation time for MixML-SEM with the correct number of clusters was 1.8 min for Step 1, 0.4 s for Step 2 and 4.6 min for Step 3 (with 50 random starts). Note that the average computation time of Step 1 was mainly influenced by the number of groups: 1.5 min for 48 groups, 2.0 min for 96 groups. For Step 3, the computation time varied depending on all simulation conditions. The lowest average was 0.4 min for “easy” conditions (e.g., *K* = 2, β = 0.4, with only large groups and fixed within-group samples), while the highest average was 24.5 min for “hard” conditions (e.g., *K* = 4, β = 0.2, with only small groups and random within-group samples).

#### Results

##### MixML-SEM results

###### Recovery of the measurement model

For the invariant loadings (excluding the fixed marker variable loadings), on average across simulated data sets, the estimated values amounted to 0.775 (*SD* = 0.005) and 0.634 (*SD* = 0.007) for the two reliability levels, closely matching the data generating values of $\sqrt{0.6}$ and $\sqrt{0.4}$, respectively. Recall that, for the non-invariant loadings, only the mean and variance of the random effects are estimated as parameters. In case of high reliability, on average across simulated data sets (and the two non-invariant loadings), the estimated mean of the loadings was 0.775 (*SD* = 0.021), with a variance of 0.100 (*SD* = 0.012). For the low reliability conditions, the estimated mean was 0.634 (*SD* = 0.021) with the same variance of 0.100 (*SD* = 0.013). This closely matches the data generating values for the random loadings' distribution, with a mean of $\sqrt{0.6}$ or $\sqrt{0.4}$ and a variance of 0.1. No large effects were found for the other manipulated factors.

For the non-invariant loadings, we also assessed the accuracy of the group-specific loading estimates derived from the random effects. To this end, we computed the Root Mean Squared Error (RMSE):


(18) $$\begin{array}{l} RMSE_{\lambda }=\sqrt{\frac{\sum_{g=1}^{G}{\sum_{j=1}^{J}\left({\hat{\lambda }}_{jg}-\lambda_{jg}\right)}^{2}}{GJ}} \end{array}$$

where λ_*jg*_ is the true group-specific loading of item *j*, and ${\hat{\lambda }}_{jg}$ is the corresponding estimate. Only non-invariant loadings are included in this computation. On average across all data sets, *RMSE*_λ_ amounted to 0.079 (*SD* = 0.041). It was mainly influenced by group sizes and whether within-group samples were fixed or random. In particular, for fixed within-group samples, the largest average *RMSE*_λ_ was 0.080 when *N*_*g*_ = 25 for all groups, and the smallest average was 0.019 when *N*_*g*_ = 200 for all groups. For random within-group samples, the largest average *RMSE*_λ_ was 0.162 when *N*_*g*_ = 25 for all groups, decreasing to 0.064 when *N*_*g*_ = 200 for all groups.

For the invariant unique variances, the mean parameter values were 0.395 (*SD* = 0.005) and 0.593 (*SD* = 0.008) for the two reliability levels, closely matching the data generating values of 0.4 and 0.6, respectively. For the non-invariant, random unique variances, the estimated means were, on average, equal to −0.968 and −0.563 (*SD* = 0.025) on the log scale, closely matching the data generating values of −0.948 and −0.542. Again, no large effects were found for the other manipulated factors. We also evaluated the group-specific estimates derived from the random effects, with a similar RMSE as for the non-invariant loadings. Across all data sets, *RMSE*_θ_ amounted to 0.073 (*SD* = 0.024) on average, mainly affected by group sizes and whether within-group samples were fixed or random: In fixed conditions, the average *RMSE*_θ_ was 0.089 with *N*_*g*_ = 25 for all groups, and 0.026 with *N*_*g*_ = 200 for all groups. In random conditions, *RMSE*_θ_ was on average 0.113 when *N*_*g*_ = 25 for all groups and 0.058 when *N*_*g*_ = 200 for all groups.

To conclude, the invariant measurement parameters and the random effects for the non-invariant ones were recovered very well. As expected, for the non-invariant parameters, the group-specific estimates derived from the random effects were biased for smaller groups, especially in case of random within-group samples, indicating the shrinkage of the group-specific estimates toward the mean.

###### Sensitivity to local maxima

To check how often (Step 3 of) MixML-SEM converged to a local maximum, we compared the final log-likelihood to a “proxy” of the global maximum likelihood solution. This proxy was obtained by starting Step 3 with the true clustering instead of a random clustering (Perez Alonso et al., 2024). When the final log-likelihood (i.e., the highest one resulting from the 50 random starts) was more than 0.001 smaller than the log-likelihood of the proxy, it was considered a local maximum. By this definition, MixML-SEM converged to a local maximum for 0.1% of all data sets.

###### Cluster recovery

To evaluate the cluster recovery, we made use of the Adjusted Rand Index (ARI; Hubert and Arabie, 1985), which measures the agreement between two partitions, where 1 indicates complete agreement and 0 the level of agreement one would find for two random partitions. For computing the ARI, we transformed the estimated cluster memberships into a hard partition, by assigning each group to the cluster with the highest classification probability, and then compared it to the true clustering. To get a better feeling of how many of the groups were clustered (in)correctly, we also evaluated the correct clustering rate (%CC), defined as the percentage of correctly clustered groups for each data set. To evaluate whether a worse cluster recovery concurred with a higher classification uncertainty, we inspected the highest classification probability for each group (${\hat{z}}_{gk}^{\max}$), where this probability being smaller than 1 would indicate uncertainty. Hence, classification uncertainty was quantified as $\left(1-{\hat{z}}_{gk}^{\max}\right)$ for each group, which was then averaged across groups per data set. The ARI, %CC, and classification uncertainty are reported in Table 1.

**Table 1:** MixML-SEM cluster recovery.

**Factor**	**Level**	**ARI**	**%CC**	**Uncertainty**
*G*	48	0.735 (0.339)	0.876 (0.192)	0.088 (0.132)
	96	0.755 (0.319)	0.887 (0.182)	0.092 (0.137)
*K*	2	0.805 (0.280)	0.937 (0.104)	0.053 (0.075)
	4	0.685 (0.363)	0.826 (0.230)	0.127 (0.167)
Large *N*_*g*_	100	0.714 (0.345)	0.864 (0.200)	0.098 (0.142)
	200	0.776 (0.310)	0.898 (0.172)	0.083 (0.126)
Small *N*_*g*_	25	0.698 (0.358)	0.856 (0.206)	0.113 (0.148)
	50	0.791 (0.291)	0.907 (0.162)	0.067 (0.115)
Small groups proportion	0	0.899 (0.186)	0.961 (0.083)	0.021 (0.033)
	0.25	0.839 (0.228)	0.938 (0.102)	0.052 (0.052)
	0.5	0.779 (0.277)	0.911 (0.130)	0.084 (0.084)
	0.75	0.693 (0.345)	0.858 (0.194)	0.122 (0.139)
	1	0.512 (0.412)	0.738 (0.268)	0.171 (0.216)
β	0.2	0.571 (0.397)	0.785 (0.240)	0.175 (0.171)
	0.3	0.776 (0.288)	0.902 (0.160)	0.071 (0.110)
	0.4	0.887 (0.181)	0.957 (0.080)	0.023 (0.030)
Reliability	high	0.760 (0.321)	0.889 (0.183)	0.092 (0.135)
	low	0.729 (0.337)	0.874 (0.191)	0.088 (0.134)
Within-group samples	fixed	0.916 (0.275)	0.942 (0.193)	0.107 (0.180)
	random	0.573 (0.287)	0.820 (0.160)	0.073 (0.056)
Total		0.745 (0.329)	0.881 (0.187)	0.090 (0.135)

The average ARI, correct clustering rate (%CC), and classification uncertainty for MixML-SEM per level of each manipulated factor. The standard deviation is shown in brackets.

**Figure 3:**
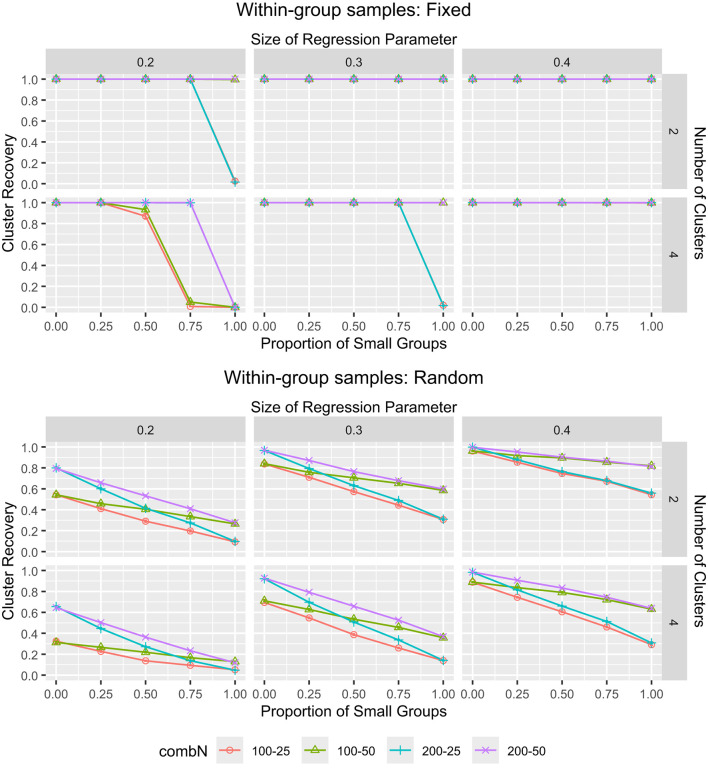
The ARI for MixML-SEM. The ARI and associated error bars for MixML-SEM in function of the within-group sample sizes for large and small groups, proportion of small groups, number of clusters, and size of regression parameters. **(Top)** Fixed within-group samples. **(Bottom)** Random within-group samples. Note that the standard errors are too small for the error bars to be clearly visible in the plots, the largest observed standard error was 0.020, corresponding to ARI values ranging from 0.850 to 0.890 for conditions with a sample size of 25 for half of the groups and 100 for the other half, *K* = 4, β = 0.2, and fixed within-group samples. “combN” refers to the combination of large and small groups.

On average, across all simulated conditions, the ARI amounted to 0.745 and the correct clustering rate was 88.1% (Table 1). To check which main and interaction effects of the manipulated factors significantly influenced the ARI, we performed an analysis of variance (ANOVA) by means of the *aov* function in R. To keep the results comprehensible, we only included two-way interaction effects. The ANOVA results table is provided in Supplementary material S2. Firstly, we see that all main effects were significant at the α = 0.01 level, and that the effects of *K*, β, small groups proportion and fixed/random within-group samples each had partial η^2^ values larger than 0.10, indicating that each of them accounted for a relatively large proportion of variance in the ARI after accounting for all other effects. From Table 1, we see that larger *G*, smaller *K*, larger groups (and a larger proportion thereof), larger β (and, thus, larger differences between clusters), higher reliability, and fixed (rather than random) within-group samples all contributed to a better recovery of the clusters. Secondly, we see that all two-way interaction effects involving β or fixed/random within-group samples were significant and that most interaction effects involving the small groups proportion or *K* were significant. The interaction between these four manipulated factors is thus interesting to inspect further and, given the inclusion of the small groups proportion, including the sample size of the small and large groups is also informative. Therefore, the interaction effect of these six manipulated factors is shown in Figure 3 for ARI. Specifically, the combination of group sizes of 200 and 50 showed the best recovery of clusters, while 100 and 25 showed the worst. In case of fewer clusters, fixed within-group samples and a β of 0.3 or 0.4, the performance was less sensitive to the group sizes. According to Steinley (2004), ARI values >0.80 indicate a good recovery of the clusters. Using this rule-of-thumb, for fixed within-group samples, the cluster recovery was good when β = 0.4, or when β = 0.3 and at least 25% of the groups were large, or when β = 0.2 and at least 50% of the groups were large. For random within-group samples, the cluster recovery was (generally) good when β = 0.4 and at least 75% of the groups were large, or when β = 0.3 and at least 75% of the groups were large with *N*_*g*_ = 200.

From Figure 3, it is clear that the cluster recovery was the worst when the proportion of small groups was 1, and that the effect of the small groups proportion was different for fixed (Figure 3, top) than for random (Figure 3, bottom) within-group samples. For fixed within-group samples, when the proportion of small groups was 1, the average ARI was 0.670, but decreasing the proportion from 1 to 0.75 already resulted in a remarkable improvement in cluster recovery, with an average ARI of 0.919. We get a better picture of what this implies in terms of correctly clustered groups by linking this to the %CC. Specifically, the %CC was 77.3%, when all groups were small and 94.2% when 75% of the groups were small. For random within-group samples, the average ARI was only 0.354 when all groups were small, which still corresponds to 70.3% of the groups being clustered correctly. Decreasing the proportion from 1 to 0.75 improved the ARI to 0.467 and the %CC to 77.5%, which is a less dramatic improvement than for the fixed within-group samples. Indeed, in the bottom panel of Figure 3, we see a more gradual improvement when the proportion of small groups decreases.

Note that, in the hard partition, it can occur that all (or most) groups are clustered into one cluster, which results in a very low ARI. In our simulations, all groups ended up in one cluster for 1,736 data sets. All of these cases occurred in fixed within-group samples, 1,348 occurred in case of four clusters, 1,547 occurred when the proportion of small groups was 1, and 1,374 occurred when this proportion was combined with β = 0.2. For the remaining 3,253 data sets with a small groups proportion of 1 in fixed within-group samples (where the groups did not end up in one cluster), the average ARI was 0.988, which indicated that the much lower ARI for a small groups proportion of 1 (as opposed to 0.75) is largely explained by the one-cluster solutions. This one-cluster issue may be explained by a high classification uncertainty. Throughout the iterative estimation process of Step 3, high classification uncertainty results in more similar cluster-specific regression coefficients, because then all groups—to some extent—affect their estimation, which is based on a weighted sum of the group-specific factor covariances with the cluster memberships serving as the weights (Perez Alonso et al., 2024). This may re-enforce the uncertainty in the next iteration. As such, it may happen that the cluster-specific regression coefficients become nearly identical, eventually resulted in one cluster after hard partitioning.

We also checked whether a worse cluster recovery (i.e., lower ARI and %CC) concurred with a higher classification uncertainty. From Table 1, we see that the main effects of the following factors on classification uncertainty were opposite to their main effects on ARI and CC%: *G*, reliability, and within-group samples. Note that the effects of *G* and reliability varied depending on the within-group samples being fixed or random. For fixed within-group samples, increasing *G* slightly reduced classification uncertainty (from 0.108 at *G* = 48 to 0.106 at *G* = 96), which concurred with a slight increase in ARI (from 0.915 to 0.917) and %CC (from 0.942 to 0.943). Classification uncertainty remained the same across the two reliability levels (0.107), as did ARI (0.916) and %CC (0.942). In contrast, for random within-group samples, classification uncertainty was lower in general, but increased with both larger *G* (from 0.069 to 0.077) and higher reliability (from 0.069 to 0.078), whereas both larger *G* and higher reliability led to a better cluster recovery (i.e., a better ARI and %CC). A possible explanation is that, in random within-group samples, additional differences were introduced across groups due to sampling fluctuations, making the groups appear more separated (even within clusters), which can lead to lower classification uncertainty, even when it leads to groups being misclassified at the same time. Conversely, a larger *G* and higher reliability help to recover the clustering, but the sampling fluctuations still lead to (slightly more) classification uncertainty. The other factors showed main effects that were consistent with expectations, with a lower classification uncertainty concurring with a better cluster recovery. Smaller *K*, larger groups, and larger β all contributed directly to a lower classification uncertainty, since less clusters imply less cluster memberships to estimate, larger groups lower the sampling fluctuations and larger β implies larger between-cluster differences that stand out over sampling fluctuations. In Supplementary material S3, Figure 1, we depicted the same interaction effect as the one we explored for the ARI. It clearly shows that classification uncertainty was generally higher for fixed within-group samples. Specifically, for fixed within-group samples, the classification uncertainty was the highest when the proportion of small groups was 1. Decreasing the proportion of small groups to 0.75 not only improved cluster recovery (as mentioned above), but also lowered the mean uncertainty from 0.252 to 0.143. Since the classification uncertainty was generally lower in random than fixed within-group samples, it does not explain poor cluster recovery in these conditions. For random within-group samples, the poor cluster recovery is instead explained by sampling fluctuations and the fact that sampling fluctuations tend to be larger especially for smaller groups, which means that the observed data are less representative of the larger population. Therefore, a larger group size is required for better cluster recovery in random within-group samples.

###### Regression parameter recovery

To evaluate the recovery of the regression parameters, we computed the Root Mean Squared Error (RMSE) for each regression parameter (i.e., for β_1_, β_2_, β_3_, and β_4_) separately:


(19) $$\begin{array}{l} RMSE_{\beta }=\sqrt{\frac{\sum_{k=1}^{K}{\left({\hat{\beta }}_{k}-\beta_{k}\right)}^{2}}{K}} \end{array}$$

where β_*k*_ and ${\hat{\beta }}_{k}$ are the true and estimated values of the regression coefficient in cluster *k*, respectively. The main effects of the manipulated factors on each *RMSE*_β_ are summarized in Table 2. On average, *RMSE*_β_ was 0.033, 0.030, 0.028, and 0.040 for β_1_, β_2_, β_3_, and β_4_, respectively. Note that differences in β_1_ and β_2_ were manipulated across all conditions while differences in β_3_ and β_4_ were only manipulated when *K* = 4. When *K* = 2, the average *RMSE*_β_ was 0.022, 0.021, 0.017, and 0.028 for β_1_, β_2_, β_3_, and β_4_, respectively; when *K* = 4, the average *RMSE*_β_ was 0.044, 0.040, 0.039, 0.052 for these regression coefficients respectively. Thus, the parameter recovery of β_1_ and β_4_ was generally worse than that of β_2_ and β_3_. This is consistent with previous findings that the further away the parameters are from the exogenous latent variables (i.e., *F*1 and *F*2), the worse their recovery (Devlieger and Rosseel, 2017; Guenole and Brown, 2014; Perez Alonso et al., 2024). In our model, β_4_ is not directly connected to any of the exogenous factors and the estimation of β_1_ relies on that of β4 since they both pertain to regression effects on the same variable (*F*4). This may explain why β_1_ and β_4_ were less accurately recovered than β_2_ and β_3_.

**Table 2:** MixML-SEM regression parameter recovery: *RMSE*_β_.

**Factor**	**Level**	**β_1_**	**β_2_**	**β_3_**	**β_4_**
*G*	48	0.038 (0.055)	0.035 (0.050)	0.032 (0.049)	0.045 (0.051)
	96	0.028 (0.040)	0.026 (0.037)	0.024 (0.035)	0.035 (0.038)
*K*	2	0.022 (0.030)	0.021 (0.028)	0.017 (0.024)	0.028 (0.026)
	4	0.044 (0.059)	0.040 (0.054)	0.039 (0.053)	0.052 (0.056)
Large *N*_*g*_	100	0.038 (0.052)	0.035 (0.047)	0.032 (0.044)	0.044 (0.048)
	200	0.028 (0.043)	0.026 (0.041)	0.024 (0.041)	0.036 (0.042)
Small *N*_*g*_	25	0.040 (0.059)	0.037 (0.054)	0.033 (0.052)	0.045 (0.056)
	50	0.026 (0.031)	0.024 (0.030)	0.023 (0.030)	0.034 (0.031)
Small groups proportion	0	0.016 (0.017)	0.014 (0.017)	0.014 (0.016)	0.026 (0.017)
	0.25	0.018 (0.022)	0.016 (0.020)	0.016 (0.020)	0.028 (0.020)
	0.5	0.024 (0.030)	0.021 (0.027)	0.021 (0.027)	0.032 (0.030)
	0.75	0.035 (0.045)	0.032 (0.041)	0.030 (0.040)	0.042 (0.045)
	1	0.073 (0.075)	0.068 (0.069)	0.059 (0.070)	0.071 (0.073)
β	0.2	0.045 (0.058)	0.043 (0.054)	0.037 (0.054)	0.043 (0.055)
	0.3	0.030 (0.047)	0.027 (0.044)	0.026 (0.041)	0.038 (0.045)
	0.4	0.024 (0.033)	0.020 (0.028)	0.020 (0.027)	0.038 (0.032)
Reliability	high	0.030 (0.041)	0.027 (0.039)	0.025 (0.038)	0.036 (0.039)
	low	0.037 (0.054)	0.034 (0.049)	0.031 (0.047)	0.043 (0.051)
Within-group samples	fixed	0.017 (0.026)	0.015 (0.026)	0.012 (0.023)	0.026 (0.023)
	random	0.049 (0.058)	0.046 (0.053)	0.044 (0.051)	0.054 (0.056)
Total		0.033 (0.048)	0.030 (0.044)	0.028 (0.043)	0.040 (0.045)

The average Root Mean Squared Error (RMSE) for MixML-SEM per level of each manipulated factor for each of the four estimated regression parameters. The standard deviation is shown in brackets.

**Figure 4:**
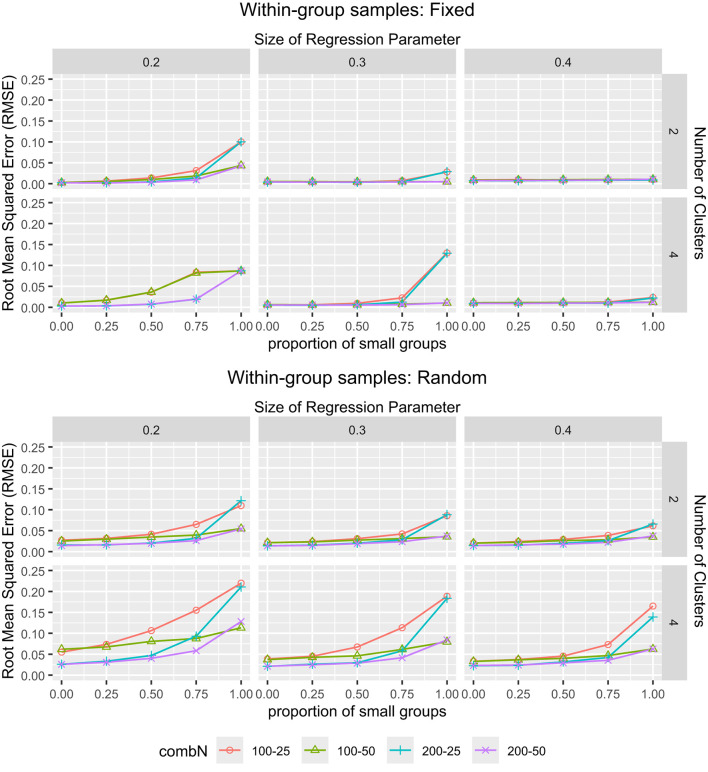
*The RMSE*_β_ for MixML-SEM. *The RMSE*_β1_ for MixML-SEM in function of the within-group sample sizes for large and small groups, proportion of small groups, number of clusters, and size of regression parameters. **(Top)** Fixed within-group samples. **(Bottom)** Random within-group samples. “combN” refers to the combination of large and small groups.

Figure 4 displays the same interaction effect for *RME*_β1_ as the one previously explored for the ARI. Specifically, the largest *RMSE*_β1_ values were found when the proportion of small groups was 1, small group sample size was 25, *K* = 4, and β = 0.2, in random within-group samples. The interaction plots for *RMSE*_β2_ to *RMSE*_β4_, showing similar patterns, are provided in Supplementary material S3, Figures 2–4. The worse recovery of the regression coefficients in these conditions is explained by the worse cluster recovery in these conditions, since the estimation of the cluster-specific regression coefficients is directly affected by groups being clustered incorrectly and/or clustered with more classification uncertainty. For fixed within-group samples, the average *RMSE*_β_ values were 0.045, 0.043, 0.031 and 0.042 when all groups were small, which dropped to 0.018, 0.015, 0.012, and 0.026, respectively, when the proportion of small groups decreased from 1 to 0.75. For random within-group samples, *RMSE*_β_ was on average 0.101, 0.093, 0.088, and 0.102 when all groups were small, which dropped to 0.053, 0.049, 0.047, and 0.057, when the small groups proportion was 0.75. Other than that, we see that larger *K*, smaller β values, and smaller group sizes (i.e., the large group sample size being 100 and/or the small group sample size being 25), led to higher *RMSE*_β_ values.

##### Comparison to ML-SEM

ML-SEM estimates the MM and SM at the same time, with random effects for the four regression parameters, in addition to the random effects for the non-invariant measurement parameters. In comparison to MixML-SEM, the recovery of the measurement parameters was very similar, so we focus on the recovery of the regression coefficients. For each regression parameter, we computed the *RMSE*_β_ as follows:


(20) $$\begin{array}{l} RMSE_{\beta }=\sqrt{\frac{\sum_{g=1}^{G}{\left({\hat{\beta }}_{g}-\beta_{k}\right)}^{2}}{G}} \end{array}$$

where we compared the group-specific estimates (${\hat{\beta }}_{g}$) to the cluster-specific true values (β_*k*_) for the cluster the group truly belongs to.

On average, the *RMSE*_β_ values were 0.098, 0.096, 0.055, and 0.059 for β_1_, β_2_, β_3_, and β_4_, respectively (see Table 3). In comparison to MixML-SEM, we thus observed larger *RMSE*_β_ values, especially for β_1_ and β_2_. This is due to the random effects being normally distributed so that the derived group-specific estimates are biased toward the overall mean parameter value. Indeed, when looking at the estimated parameter values, for example, for a data set with β_1, *k* = 1_ = 0 and β_1, *k* = 2_ = 0.3, the mean of the regression coefficients across the groups belonging to cluster one (*k* = 1) was 0.138 (*SD* = 0.010), and for cluster two (*k* = 2), it was 0.171 (*SD* = 0.011). The group-specific estimates were thus close to the mean true value of β_1_ across clusters, which was 0.15.

**Table 3:** ML-SEM regression parameter recovery: *RMSE*_β_.

**Factor**	**Level**	**β_1_**	**β_2_**	**β_3_**	**β_4_**
*G*	48	0.098 (0.027)	0.096 (0.026)	0.057 (0.042)	0.060 (0.042)
	96	0.098 (0.026)	0.095 (0.026)	0.053 (0.043)	0.057 (0.044)
*K*	2	0.098 (0.028)	0.098 (0.027)	0.018 (0.016)	0.021 (0.016)
	4	0.098 (0.025)	0.093 (0.024)	0.092 (0.024)	0.097 (0.025)
Large *N*_*g*_	100	0.104 (0.024)	0.102 (0.023)	0.059 (0.044)	0.063 (0.045)
	200	0.092 (0.028)	0.089 (0.028)	0.051 (0.041)	0.055 (0.041)
Small *N*_*g*_	25	0.102 (0.029)	0.100 (0.029)	0.057 (0.045)	0.061 (0.045)
	50	0.094 (0.023)	0.091 (0.022)	0.052 (0.040)	0.057 (0.041)
Small groups proportion	0	0.076 (0.019)	0.072 (0.019)	0.043 (0.032)	0.048 (0.033)
	0.25	0.087 (0.017)	0.085 (0.016)	0.048 (0.037)	0.053 (0.037)
	0.5	0.098 (0.019)	0.095 (0.017)	0.054 (0.041)	0.058 (0.042)
	0.75	0.108 (0.021)	0.106 (0.020)	0.060 (0.045)	0.063 (0.046)
	1	0.121 (0.028)	0.120 (0.027)	0.070 (0.051)	0.073 (0.050)
β	0.2	0.081 (0.010)	0.081 (0.011)	0.047 (0.033)	0.048 (0.032)
	0.3	0.102 (0.022)	0.100 (0.022)	0.057 (0.044)	0.060 (0.043)
	0.4	0.111 (0.033)	0.106 (0.033)	0.060 (0.049)	0.068 (0.050)
Reliability	high	0.095 (0.026)	0.092 (0.025)	0.053 (0.042)	0.057 (0.042)
	low	0.101 (0.027)	0.099 (0.026)	0.057 (0.044)	0.061 (0.044)
Within-group samples	fixed	0.096 (0.029)	0.092 (0.028)	0.047 (0.046)	0.053 (0.046)
	random	0.101 (0.024)	0.099 (0.023)	0.063 (0.038)	0.065 (0.039)
Total		0.098 (0.027)	0.096 (0.026)	0.055 (0.043)	0.059 (0.043)

The average RMSE for ML-SEM per level of each manipulated factor for each of the four estimated regression parameters. The standard deviation is shown in brackets.

We see that the *RMSE*_β_ values for ML-SEM were larger with smaller *G*, smaller groups, lower reliability, and random within-group samples (Table 3), as was also the case for MixML-SEM. The only differences were the effects of factors β and *K*. Specifically, *RMSE*_β_ was larger for larger β due to the shrinkage effect toward the overall mean parameter. Since the latter is a weighted average of β and zero, it deviates more from the true value of either β or 0 in case of a larger β value. Regarding the number of clusters, for fixed within-group samples, *RMSE*_β_1__ increased with more clusters, while *RMSE*_β_2__ decreased. For β_2_, in case of more clusters, the group-specific estimates gravitate toward a larger overall mean parameter, resulting in a smaller deviation between the estimated and true value for most groups and thus in a smaller RMSE value. For example, when *G* = 48, *K* = 2, and β = 0.4, the overall mean parameter for β_2_ is the weighted average of β_2, *k* = 1_ = 0.4 and β_2, *k* = 2_ = 0, where each value applies to 24 groups. When *K* = 4, the overall mean parameter is larger, because, in that case, 36 groups (in Clusters 1, 3, and 4) have a β_2_ of 0.4, whereas only 12 groups (in Cluster 2) have a β_2_ of 0, resulting in smaller differences between estimates and true values for the former 36 groups. For β_1_, the overall mean parameter is the same as for β_2_, but its estimation is also influenced by that of β_4_, which may explain why the RMSE was slightly larger in case of more clusters. For random within-group samples, both *RMSE*_β_1__and *RMSE*_β_2__ decreased with more clusters, likely influenced by the sampling variability and the fact that the within-cluster sample size is smaller when *K* is larger. For β_3_ and β_4_, the RMSE values were larger when *K* = 4 in both fixed and random within-group samples, because they were only different across clusters in these conditions.

### Simulation study 2

In Simulation Study 2, we evaluated MixML-SEM in terms of model selection. Specifically, we ran (Step 3 of) MixML-SEM with one to six clusters for the first 10 replications of each cell of the design of Simulation Study 1, excluding conditions with “high” reliability (i.e., for a total of 4,800 data sets). Then, the number of clusters was selected based on BIC_*G*_, AIC, and CHull.

#### Results

For each data set, we verified whether the correct number of clusters was selected for MixML-SEM. BIC_*G*_ correctly selected the number of clusters for 64.8% of the data sets whereas AIC did so for 69.1% and CHull for 71%. Note that for 5.0% of the data sets, CHull did not provide a solution and they were classified as incorrect. This occurred because the “observed” single-indicator log*L* did not increase monotonically with more clusters, which is attributed to the fact that, during Step 3 of the model estimation, we maximized log*L*_η_ (Equation 13) rather than the “observed” single-indicator log*L* (Equation 15). This causes the CHull procedure to exclude the concerned models from the selection. In practice, visual inspection of the CHull plot would alleviate the problem, since a clear elbow may still be present.

Both BIC_*G*_ and AIC had a tendency to underestimate the number of clusters. Specifically, BIC_*G*_ underestimated the number of clusters for 35.1% of the data sets and selected one cluster for 26.3%. Similarly, AIC selected too few clusters for 24.4% of the data sets and selected only one cluster for 17.6%. Note that these selections of one-cluster or too-few-clusters models could not be fully explained by MixML-SEM's tendency to assign all groups to one cluster in specific conditions when using the true *K*, which would make it harder to select the correct *K*. This occurred in only 11.0% and 14.2% of the data sets for which BIC_*G*_ or AIC selected one cluster, respectively; and in only 8.7% and 11.4% of the data sets where BIC_*G*_ or AIC selected too few clusters. Since CHull selects at least two clusters, we also examined the performance of BIC_*G*_ and AIC when only considering two or more clusters, to make the comparison more fair. In this case, the overall accuracy of BIC_*G*_ increased to 74.4%, and that of AIC increased to 79.4%, which are slightly better than CHull.

The main effects of the simulated conditions on the model selection accuracy are given in Table 4. For BIC_*G*_ and AIC, larger regression coefficients, fewer clusters, a lower proportion of small groups, and random within-group samples, all contributed to a more accurate model selection. The worse performance for fixed within-group samples can only be partially explained by the occurrence of solutions where all groups were modally assigned to one cluster when using the true *K*, even though this only occurred in case of fixed within-group samples. After excluding the data sets for which one-cluster solutions occurred for fixed within-group samples (177 out of 2,400 data sets), the model selection accuracy increased to 0.630 for BIC_*G*_ (which is still lower than the BIC_*G*_ for random within-group samples), and 0.717 for AIC (which is now higher than the AIC for random within-group samples). Another potential explanation for the better performance in case of random within-group samples is that the tiny differences due to sampling fluctuations counter the tendency of BIC_*G*_ and AIC to select too few clusters. Note that having a few large groups led to a marked increase of the model selection accuracy. Specifically, for BIC_*G*_, the correct selection rate increased from 28.0% to 56.8% when the proportion of small groups decreased from 1 to 0.75. For AIC, it increased from 39.1% to 66.3%.

**Table 4:** The percentage of data sets for which the correct number of clusters for MixML-SEM was selected using BIC_*G*_, AIC and CHull, per level of each manipulated factor.

**Factor**	**Level**	**BIC*_*G*_***	**AIC**	**CHull**
*G*	48	0.591 (0.492)	0.658 (0.475)	0.691 (0.462)
	96	0.705 (0.456)	0.724 (0.447)	0.729 (0.444)
*K*	2	0.783 (0.412)	0.801 (0.400)	0.781 (0.413)
	4	0.510 (0.500)	0.579 (0.494)	0.638 (0.481)
Large *N*_*g*_	100	0.547 (0.498)	0.615 (0.487)	0.646 (0.478)
	200	0.748 (0.434)	0.765 (0.424)	0.773 (0.419)
Small *N*_*g*_	25	0.602 (0.490)	0.653 (0.476)	0.699 (0.459)
	50	0.689 (0.463)	0.725 (0.447)	0.720 (0.449)
Small groups proportion	0	0.846 (0.361)	0.809 (0.394)	0.756 (0.430)
	0.25	0.793 (0.405)	0.811 (0.391)	0.831 (0.375)
	0.5	0.722 (0.448)	0.758 (0.429)	0.815 (0.389)
	0.75	0.568 (0.496)	0.663 (0.473)	0.708 (0.455)
	1	0.280 (0.449)	0.391 (0.488)	0.424 (0.495)
β	0.2	0.334 (0.472)	0.456 (0.498)	0.551 (0.498)
	0.3	0.734 (0.442)	0.765 (0.424)	0.748 (0.435)
	0.4	0.904 (0.294)	0.871 (0.335)	0.846 (0.361)
Reliability	low	0.648 (0.478)	0.691 (0.462)	0.710 (0.454)
Within-group samples	fixed	0.600 (0.490)	0.686 (0.464)	0.718 (0.450)
	random	0.702 (0.457)	0.696 (0.460)	0.701 (0.458)
Total		0.648 (0.478)	0.691 (0.462)	0.710 (0.454)

**Figure 5:**
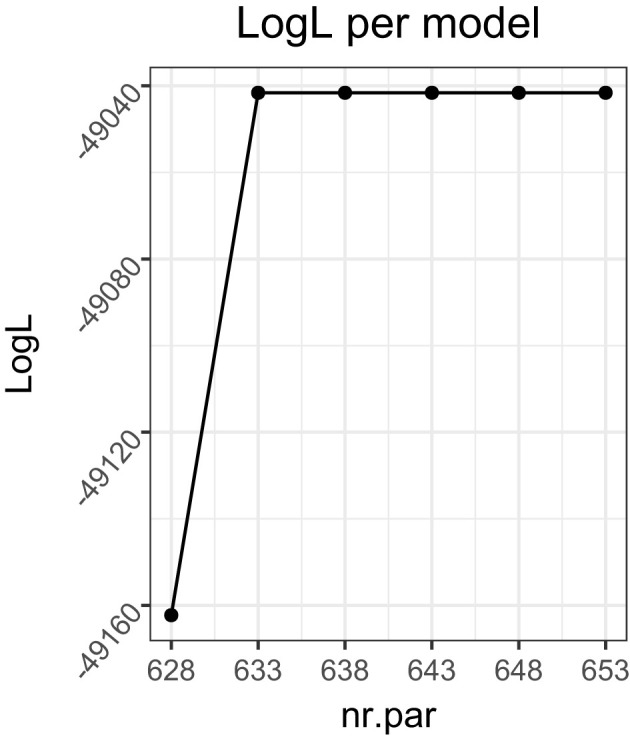
The CHull plot of an example of selecting an overly complex model for MixML-SEM based on the scree ratio's. The data set contained 48 groups, two clusters, balanced, a fixed sample of size 200 per group and β = 0.3.

The model selection performance of CHull showed similar trends to that of BIC_*G*_ and AIC, but a difference is that CHull performed slightly better for fixed within-group samples (fixed: 71.8%, random: 70.1%). For random within-group samples, the correct model selection rate increased with a larger proportion of larger groups, while, for fixed within-group samples, the best performance occurred when the proportion of small groups was 0.5 rather than 0. This may be attributed to CHull selecting overly complex models when a more complex model barely resulted in a better model fit, leading to an artificially inflated scree ratio because the denominator approaches zero (Wilderjans et al., 2013). An infinite scree ratio occurred only in fixed within-group samples (144 data sets), which is explained by the group-specific covariances being unaffected by sampling fluctuations. Also, it occurred more frequently with fewer clusters (125 data sets), more groups (81 data sets) and a smaller proportion of small groups (107 data sets). All 144 data sets with an infinite scree ratio selected an incorrect number of clusters. Recall that, in practice, researchers can visually inspect the CHull plot to identify the elbow and avoid an overly complex model being selected based on the scree ratio's alone. As an example, consider the scree ratio's and scree plot (Figure 5) for one of the simulated data sets. This data set contained two clusters, 48 groups, a fixed sample of 200 per group and β = 0.3. CHull suggests three clusters with a ratio of infinity, whereas a very clear elbow is visible for two clusters (the only elbow in the plot), which is captured by the second largest scree ratio (8.292e+12). Given the impracticality of checking the CHull plot for each simulated data set, we examined the second largest scree ratio for all data sets with a maximal scree ratio of infinity and an incorrect model selection. By selecting the model with the second largest scree ratio, the number of clusters was correctly identified for all these data sets. In this way, the model selection accuracy for fixed within-group samples improved from 41.3%, 77.5%, 87.1%, 85.2%, and 67.9% to 41.9%, 78.3%, 88.8%, 89.8% and 90.2% for the proportion of small groups ranging from 1 to 0, respectively, indicating an improvement in model selection accuracy with a larger proportion of larger groups.

### Conclusion

We assessed the performance of MixML-SEM and compared it to ML-SEM when the measurement non-invariances were correctly specified. When the true number of clusters was specified (Simulation Study 1), MixML-SEM performed well when the cluster separation was sufficiently large (for example β = 0.3) and/or when more large groups were involved, outperforming ML-SEM in terms of regression parameters recovery. The results suggest that the required group sizes for reliable performance depend on the degree of cluster separation and whether one wants to learn about structural relations for the specific within-group samples (fixed within-group samples) or aims to make inferences about a larger population with the groups (random within-group samples). In more challenging conditions with very small cluster differences (e.g., β = 0.2), a minimum of 50% group sizes of 100 was needed to achieve good performance under the current model setup in case of fixed within-group samples, while only 25% or even 0% of large groups was needed with better cluster separation (e.g., β = 0.3 *or* 0.4). For random within-group samples, larger group sizes (e.g., 200 or larger) and/or a larger proportion of large groups were required. Thus, applied researchers should keep in mind that, when (some) group sizes are smaller than 100, small differences in structural relations may not be captured, especially when they are masked by sampling fluctuations and one wants to draw conclusions about the larger population if the samples are not representative.

Despite difficulties in recovering the clustering when having only small groups combined with a low cluster separation, we observed a notable improvement in the performance of MixML-SEM when more larger groups were included alongside the small groups. This confirms the main advantage of MixML-SEM: combining information from multiple groups within a cluster leads to a better regression parameter (and cluster) recovery.

In contrast, in ML-SEM, the group-specific regression parameter estimates, derived from the random effects, were biased toward the overall mean parameter value. Hence, if researchers compare the group-specific estimates to draw conclusions about differences and similarities in structural relations, the differences are obfuscated due to the shrinkage bias. If they would opt for a mixture clustering based on such biased estimates to make the comparisons, the clustering will not be accurate either. Even when the clustering would be accurately recovered, the comparison of the corresponding cluster-specific regression coefficients would still be affected by the bias.

When it comes to selecting the number of clusters for MixML-SEM (Simulation Study 2), we conclude that BIC_*G*_, AIC, and CHull have comparable performance. BIC_*G*_ and AIC tend to be more conservative, preferring models with fewer clusters, but they have the advantage over CHull that they can select one cluster. However, violations of distributional assumptions may lead to overselection in case of the BIC (and AIC) (e.g., Bauer, 2007; McNeish and Harring, 2017), making CHull potentially more suitable for empirical data. In CHull, an artificially inflated scree ratio may lead to overselection as well, but we can consider the two best models and/or visually inspect the CHull plot to confirm the presence of an elbow. Therefore, in empirical practice, we recommend combining BIC_*G*_, AIC, and CHull, along with visual inspection of the CHull scree plot.

## Empirical application

In this section, we demonstrate the empirical value of MixML-SEM using data from Dejonckheere et al. (2022), where they investigated how the perceived social emotion norm—i.e., the social pressure to feel positive, and not to feel negative—relates to people's subjective wellbeing across 40 countries and territories. For this illustration, we focus on the relation between participants' perceived social pressure to be happy and life satisfaction. The perceived social pressure to be happy was assessed using the nine-item Social Expectancies about Happiness Scale (SEHS; Dejonckheere et al., 2022), with items such as “I often feel a great deal of pressure from those around me to feel Happy.” Participants rated each item on a Nine-point Likert scale ranging from strongly disagree (one) to strongly agree (nine). Life satisfaction was assessed with the Five-item Satisfaction with Life Scale (SWLS; Diener et al., 1985), including items like “The conditions of my life are excellent,” rated on a Seven-point Likert scale from strongly disagree (one) to strongly agree (seven). Dejonckheere et al. (2022) conducted multilevel regression analysis with random intercepts and slopes, based on mean scores computed for each construct. They found a fixed effect of −0.05 and a random effect standard deviation of 0.11 for the regression coefficient of SEHS on SWLS, indicating substantial variability of this relations across the countries. Specifically, five countries had significantly positive relations between SEHS and SWLS, 10 had significantly negative relations, and 25 had null-relations. They used the world happiness index (WHI) as a country-level predictor to explain the between-country variability and concluded that SEHS was linked to lower SWLS in high WHI countries (β = − 0.08).

By using mean scores, Dejonckheere et al. ignored (1) that the constructs are measured by items containing measurement error, and (2) that this measurement may be non-invariant across countries, both of which can result in biased regression estimates. Moreover, as mentioned in the Introduction, multilevel modeling is not ideal for identifying specific differences between the 40 countries. Specifically, it requires 780 pairwise comparisons of country-specific regression estimates, which are biased by the shrinkage toward the overall mean—in addition to the bias by measurement error and potential non-invariances.

To solve all these issues, we applied MixML-SEM to the data. After removing observations with missing data for the variables of interest, we retained a sample of 6,775 participants from 40 countries. The mean structure was removed by centering the items per group. Before applying MixML-SEM, we evaluated MI for each construct separately using ML-CFA in Mplus. We first examined the variances of the measurement parameters when all of them were set to be random to see which ones have the largest variance. Then, we estimated several ML-CFA models, each time adding a random measurement parameter in the order of the magnitude of the random effect variance in the model where all measurement parameters were random. We compared these models by means of the deviance information criterion (DIC; Spiegelhalter et al., 2002), which balances model fit and complexity for Bayesian models, based on the posterior mean estimate. The MI testing revealed three non-invariant factor loadings (of items 1, 6, and 9) for SEHS, in addition to the need to include residual covariances between certain items (items 1 and 2, and items 7 and 8), and no non-invariant loadings for SWLS, with both constructs modeled with random unique variances and factor variances. The testing procedure and final model specification for both constructs (Step 1 of MixML-SEM) can be found in Supplementary material S4.

**Figure 6:**
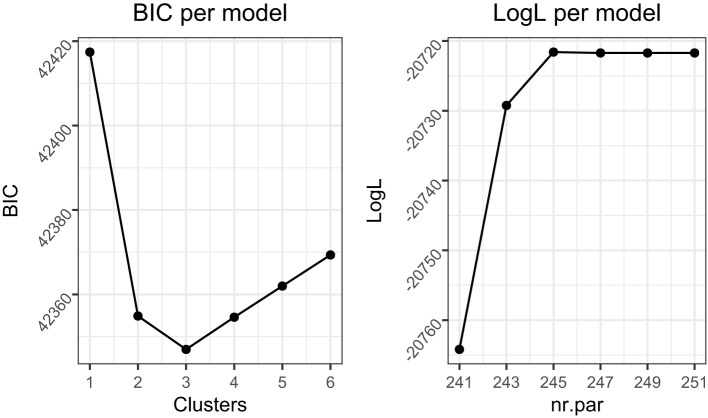
The BIC_*G*_ and CHull plot (based on the logL as a function of the number of parameters) for models with 1 to 6 clusters for the empirical data.

Given that we do not know the true underlying number of clusters, we ran (Step 3 of) MixML-SEM with one to six clusters. The BIC_*G*_ (Figure 6, left) suggests that the model with three clusters provides the best model fit, and CHull (Figure 6, right) also suggests three clusters (i.e., the plot levels off completely after three clusters). Therefore, we present the results for the Three-cluster model (Table 5).

**Table 5:** The clustering of the countries based on the regression parameters between social pressure to be happy and life satisfaction (3-cluster model).

**Model**	**Countries**
Cluster 1 (8)	Chile (0.73), China, Hong Kong, Nigeria, Pakistan, Senegal, Uganda (0.84), Ukraine (0.83)
Cluster 2 (15)	Brazil (0.88), Costa Rica (0.89), England, France, Germany, Latvia, Macedonia, Netherlands, Northern Ireland, Peru (0.66), Philippines, Poland, Scotland (0.70), South Korea (0.51), Wales
Cluster 3 (17)	Australia, Belgium, Canada, Colombia (0.85), Estonia (0.71), Italy, Japan (0.86), Malaysia, New Zealand, Portugal (0.84), Singapore (0.84), Slovakia (0.74), South Africa, Spain, Thailand (0.89), Turkey, USA

The countries with a ${\hat{z}}_{gk}$ lower than 0.90 are presented along with their corresponding posterior probabilities, enclosed in parentheses. For those countries with classification uncertainty, each consisted of a mix of either Cluster 1 or Cluster 2 with Cluster 3. For example, South Korea had the largest classification uncertainty: ${\hat{z}}_{gk}=0.51$ for Cluster 2 and 0.49 for Cluster 3, and Peru: ${\hat{z}}_{gk}=0.66$ for Cluster 2 and 0.34 for Cluster 3.

The Three-cluster model consists of Cluster 1 with a regression coefficient of 0.184 and including 8 countries, Cluster 2 with a coefficient of −0.254 and comprising 15 countries, and Cluster 3 with a coefficient of −0.038 and including 17 countries. Geographically, most European countries were classified in either Cluster 2 or Cluster 3 except for Ukraine (${\hat{z}}_{gk}=0.83$ for Cluster 1 and 0.17 for Cluster 3). Asia was also mainly distributed across two clusters, Cluster 1 and Cluster 3, except for Philippines and South Korea. South Korea was classified with a high classification uncertainty, however (${\hat{z}}_{gk}=0.51$ for Cluster 2 and 0.49 for Cluster 3). Of the four African countries, three were classified into Cluster 1 and one (South Africa) in Cluster 3. For South-American countries, all of them exhibited a classification uncertainty >0.1 (each country comprised a mix of either Cluster 1 or Cluster 2 with Cluster 3). For North America, Canada and the USA were in Cluster 3, whereas Costa Rica was in Cluster 2. For Oceania, both Australia and New Zealand were in Cluster 3.

To some extent, our findings resemble those of Dejonckheere et al. (2022), where five countries showed positive relations (Senegal, Hong Kong, Nigeria, Pakistan, and China, all found in Cluster 1 of MixML-SEM), 10 negative (Northern Ireland, Peru, Latvia, Philippines, France, Macedonia, Germany, Netherlands, England, Poland, all found in Cluster 2), while the remaining 25 were close to 0 (17 countries in Cluster 3, three in Cluster 1, and five in Cluster 2). Note that the classification by Dejonckheere et al. (2022) was solely based on whether the regression coefficient was significantly positive or negative (disregarding the actual values of the coefficient), whereas, with MixML-SEM, we assume all countries in the same cluster to have the same values for the regression coefficients.

**Figure 7:**
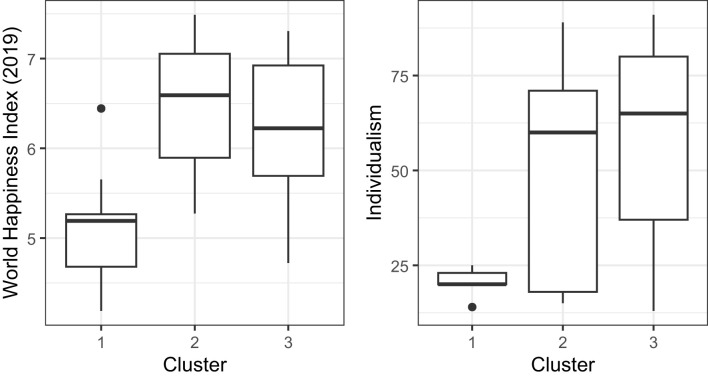
The WHI and individualism scores for the three clusters obtained by MixML-SEM.

To explore the potential influence of country-level predictors on the relation between social pressure to be happy and life satisfaction, we examined two variables: World happiness index (WHI; as in Dejonckheere et al., 2022) and individualism (from Hofstede, 2001). Figure 7 (left) presents boxplots of WHI scores for the Three-cluster model. Countries in Cluster 1 generally exhibited lower WHI values compared to the other clusters, with the exception of Chile. Clusters 2 and 3 had relatively higher WHI values, but there were exceptions (e.g., Macedonia, in Cluster 2, and South Africa, in Cluster 3, have a low WHI). In conclusion, there was considerable overlap between the three clusters, suggesting that WHI may not be the only predictor of the relation. Note that the interaction effect found by Dejonckheere et al. (2022), where SEHS was linked to lower SWLS in high WHI countries (β = −0.08), was also a weak one. To further examine whether this relation depends on individualism, Figure 7 (right) presents boxplots of the individualism scores for the three clusters, which was available for 31 of the countries. Overall, countries in Cluster 1 exhibited lower individualism, followed by countries in Clusters 2 and 3. However, the overlap between the clusters (especially between Clusters 2 and 3) suggests that other variables may also contribute to the relation between social pressure to be happy and life satisfaction.

In summary, MixML-SEM revealed cross-national differences in the relation between social pressure to be happy and life satisfaction, captured by assigning the 40 countries to three clusters. Compared to the multilevel regression analysis, MixML-SEM allowed us to avoid a large number of pairwise comparisons of biased group-specific regression estimates, while taking the measurement error and the measurement non-invariances into account.

## Discussion

MixML-SEM is a novel method for comparing structural relations between latent variables across many groups, while parsimoniously addressing potential measurement non-invariances with random effects. Specifically, after accounting for measurement non-invariances with ML-CFA, MixML-SEM uses mixture clustering to gather groups with equivalent structural relations, thereby reducing the need for pairwise comparisons of the relations. For instance, in the empirical example on social pressure to be happy and life satisfaction, comparing only three cluster-specific regression coefficients efficiently exposed the differences among 40 countries.

One would expect the parsimonious measurement model of MixML-SEM to be especially advantageous in case of (some) small groups. Small groups are more prone to sampling fluctuations, however. The simulation study demonstrated a strong effect of the fixed vs. random within-group samples on MixML-SEM's performance. Thus, a critical question is whether the analysis aims to capture clusters regarding the regression relations in the observed within-group samples or regarding the relations in the larger within-group populations. Since smaller samples are more vulnerable to sampling variability, the observed data are less representative of the population, especially when the underlying population is relatively large. Thus, when applying MixML-SEM to empirical data on small groups, researchers should be cautious when generalizing the captured differences in structural relations to larger within-group populations, unless there is a strong justification that the observed samples are representative (for instance, when within-group samples are obtained within stratified sampling). Unlike ML-SEM, MixML-SEM does not adopt the multilevel approach for the structural model, which avoids comparing biased group-specific regression estimates derived from random effects. The added value of MixML-SEM over ML-SEM was demonstrated in simulation studies. Of course, the simulation studies were limited to conditions where a clustering was indeed underlying the structural relations. Future research could evaluate how MixML-SEM performs when this is not the case. For instance, it would be interesting to see how many and which clusters are captured with MixML-SEM when the differences in structural relations are in fact gradual and normally distributed.

Furthermore, in our simulation studies, we examined a model with four latent variables and four regressions among them, whereas, in the empirical study, we applied MixML-SEM to cluster on only one regression coefficient. In practice, more variables and thus more relations can be involved. Depending on the research question and the structural model, researchers can choose to cluster all relations together or separately. Note that clustering on multiple relations may require more clusters to capture all the differences, whereas clustering on each regression coefficient separately does not allow for one relation to be estimated conditional on another one. Note that the advantage of MixML-SEM is greater in case of more complex structural models, where multiple relations may vary across clusters. That is, a clustering based on one regression coefficients might also be found by ordering the group-specific relations in terms of their direction and size, but a clustering based on multiple regression coefficients is much harder to find based on comparisons of group-specific coefficients. When clustering groups on multiple relations, it would be interesting to combine MixML-SEM with hypothesis testing on the cluster-specific regressions to determine which relations are significantly different across which clusters.

A key feature of MixML-SEM is its stepwise estimation process, which should provide robustness against local model misspecifications, as was demonstrated for the SAM approach in general (Rosseel and Loh, 2024). In contrast, simultaneous estimation of all parameters may allow misfit or uncertainty in one part of the model (e.g., the structural model) to propagate and distort estimation in another part (e.g., the measurement model), and vice versa. Future research could investigate the robustness of MixML-SEM against different forms of measurement model misspecification. This could involve scenarios such as disregarding cross-loadings or residual covariances between items, or ignoring measurement non-invariances. In the first step of MixML-SEM, the measurement model is estimated per latent variable, which corresponds to the “measurement block approach” recommended by Rosseel and Loh (Rosseel and Loh, 2024). This approach drastically reduces computation time, but requires a sufficient number of (strong) indicators for each factor. It may thus be infeasible or inadvisable when the number of indicators is insufficient and/or when their reliability is low. In such cases, the ML-CFA needs to be estimated for all (or some) latent variables simultaneously (which corresponds to combining them in one measurement block), before continuing with the estimation of MixML-SEM as usual. In the future, it would be valuable to compare the performance of MixML-SEM when starting from one vs. multiple measurement blocks in different conditions and under different misspecifications.

The stepwise approach allows for a lot of flexibility, such as using a different estimator in each step. In particular, Bayesian estimation was applied in Step 1 for estimating ML-CFA, whereas maximum likelihood estimation was applied in Steps 2 and 3. Existing mixture-based SEM methods, like multilevel mixture SEM in Mplus (where “multilevel” indicates that the mixture operates at the group level), lack such flexibility. In fact, multilevel mixture SEM does not support including random loadings for the measurement model due to the unavailability of Bayesian estimation for this model type. Consequently, differences in the measurement model are either disregarded or captured by the same clustering along with the differences in structural relations. This limitation undermines the ability to disentangle different sources of variability across groups, making it impossible to cluster on the structural relations specifically.

The stepwise estimation also facilitates extensions of the method. One possible extension involves replacing the first step by alternative approaches for handling measurement non-invariances. In MixML-SEM, we used the multilevel approach to account for measurement non-invariances, but an interesting alternative is the Multigroup Bayesian CFA approach with approximate measurement invariance (Muthén and Asparouhov, 2013) which captures differences in measurement parameters by small-variance priors. The key difference between the two approaches lies in their underlying assumptions. For the invariant measurement parameters, ML-CFA assumes exact invariance, meaning identical parameters across all groups, whereas the non-invariant measurement parameters are captured with random effects with an unrestricted variance. Especially when dealing with a large number of groups, achieving exact invariance may not always be realistic, not even for some of the measurement parameters. Approximate invariance allows for small differences across groups for many, or even all, of the measurement parameters, where these differences are kept small by restricting their variance. The fact that the differences across groups are kept small maintains the comparability of structural relations across groups. In the future, it would be interesting to investigate the interplay between exact measurement invariance, approximate measurement invariance, and measurement non-invariance within the Mixture Multigroup/Multilevel SEM framework.

Currently, MixML-SEM combines Bayesian and maximum likelihood estimation, assuming continuous items. In empirical practice, we often encounter ordinal data like questionnaire items with a specific number of response categories. Having enough response categories (say five or more) allows for the items to be considered as continuous (Dolan, 1994), but binary items or items with three or four response categories are common (e.g., in the Programme for International Student Assessment). Therefore, adapting the method to ordinal data would be another useful extension. To this end, the first step of MixML-SEM, using Bayesian estimation, should be adjusted to deal with ordinal data, whereas the rest of the estimation procedure remains unchanged, since the factor scores derived from the first step are still continuous.

As our primary focus was on comparing structural relations, our current approach disregarded the mean structure (i.e., the data were centered per group). Another possible extension is to incorporate the mean structure into the model. In addition to clustering based on regressions, we could also cluster groups based on their factor means if this is relevant to the research question. Note that comparing factor means across (clusters of) groups requires a higher level of MI—that is, at least (partial) scalar invariance—and thus a modification to the measurement model as well. Differences in factor means and structural relations will then be captured by the clusters, whereas differences in the measurement model are captured by random intercepts and/or random loadings. This combination of mixture modeling and random effects cannot be attained by existing mixture SEM methods in Mplus, because they do not allow for the required Bayesian estimation (as mentioned above).

To conclude, MixML-SEM provides a parsimonious way for accounting for measurement non-invariance (with random effects) while comparing structural relations across many groups (with mixture modeling). The combination of random effects and mixture modeling is handled in a stepwise manner, building on the SAM approach. This stepwise estimation approach is very flexible, which makes it easy to extend the method in many ways, including the use of alternative methods for addressing measurement non-invariances. MixML-SEM is thus not only a novel method for comparing structural relations among many groups, but also an important step toward a realm of possibilities for handling different types of measurement non-invariance while clustering the groups on their structural relations.

## Data Availability

The datasets presented in this study can be found in online repositories. The names of the repository/repositories and accession number(s) can be found below: https://osf.io/rtp78/.
