# Supplementary material for: Mixture Multilevel SEM vs. Multilevel SEM for comparing structural relations across groups in presence of measurement non-invariance
Source: Front Psychol. 2025 Jul 28;16:1463790. doi: 10.3389/fpsyg.2025.1463790 (PMC12337785; doi:10.3389/fpsyg.2025.1463790)
Supplement: Supplementary file 1 [file Supplementary_file_1.docx]

**Supplementary Material**

S1. MixML-SEM algorithm

S2. ANOVA table with all main effects and two-way interaction effects of the manipulated factors on ARI

S3. Supplementary figures

Fig 1. Interaction effects on classification uncertainty

Fig 2 - Fig 4. Interaction effects on ${RMSE}_{\beta}$ for $\beta_{2}$, $\beta_{3}$, $\beta_{4}$

S4. Measurement invariance testing and final syntax for empirical application on social pressure to be happy and life satisfaction

**S1. MixML-SEM algorithm**

The algorithm of MixML-SEM consists of three steps. Step 1 involves estimating the measurement model with ML-CFA. An intermediate Step 2 obtains group-specific factor covariances using the single-indicator approach. Step 3 estimates the structural model with the mixture clustering of the groups. Below is a detailed presentation of each step.

**Step 1: ML-CFA with measurement non-invariances**

In Step 1, a ML-CFA is estimated per latent variable using the Bayes estimator with default, non-informative priors by means of Mplus and the R-package MplusAutomation (Hallquist & Wiley, 2018). The default seed value is zero for the random draws in the Markov chain Monte Carlo (MCMC) chains in Mplus. For each latent variable, the following parameters need to be defined at the within-level in the Mplus syntax:

1. Factor loadings ($\boldsymbol{\lambda}_{g}$) need to be at least partially invariant for valid comparisons of structural relations. Here, users can specify the invariant loadings (e.g., “fw BY x1 x4-x5” where loadings of items 1, 4, and 5 are invariant for the factor) and non-invariant loadings ($\lambda_{jg}$) with random effects (e.g., “s2-s3 | fw BY x2-x3” where the loadings of items 2 and 3 are non-invariant for the factor). The random loadings are assumed to be normally distributed. By default, the marker variable approach is used, where the first invariant loading of each factor is set to 1 to set the scale of the latent variable;

2. Unique variances ($\boldsymbol{\Theta}_{g}$) can be fully invariant, partially invariant, or fully non-invariant. By default, they are estimated as invariant in Mplus. Thus, if non-invariant unique variances ($\Theta_{jg})$ are needed, the log of the variance is instead modeled with a normal distribution (e.g., “logx2 | x2; logx3 | x3” for non-invariant unique variances of items 2 and 3);

3. The factor variance is invariant by default in Mplus, but can also be specified as non-invariant (e.g., “logv | fw”).

Additionally, in MixML-SEM, intercepts are specified as 0 by default, since the observed scores of each item are centered within each group. Also note that measurement parameters should be specified as invariant or non-invariant according to the results of MI testing performed prior to the application of MixML-SEM. Note that including random effects for invariant parameters may not hurt parameter estimates, but it can increase computation time, especially for larger data sets. Upon estimating the measurement model for each factor, the posterior distributions of the factor are appended to the data file. These values can subsequently be used in Step 2.

**Step 2: Single-indicator approach to obtain group-specific factor covariances**

In Step 2, the factor scores (i.e., posterior means) resulting from Step 1 serve as the single “observed” indicator of the factor. The group-specific factor covariances based on the single-indicator data are computed by means of Equations (7) – (9).

**Step 3: Structural model with mixture clustering of the groups**

In Step 3, an EM algorithm is used to cluster the groups on their structural relations, using their group-specific factor covariances (resulting from Step 2) as input. In the E-step, the algorithm (re-)estimates the expected cluster memberships for all groups. In the M-step, it maximizes the unknown parameters given the expected cluster memberships obtained from the E-step. The E- and M-steps are iterated until the change in log-likelihood between iterations becomes sufficiently small. By default, this is considered to be when the change in log-likelihood is less than $1\times{10}^{-6}$ or when the number of iterations reaches 10,000.

To initialize the EM algorithm, we begin with a random clustering of the groups. The default setting employs hard partitioning, where cluster membership is either 0 or 1, and each cluster contains at least one group. Since the obtained results may depend on the initial values for the clustering, a multi-start procedure is used (e.g., 50 random starts) to prevent convergence to local maxima. For further technical details of Step 3, readers are advised to consult Appendix A of Perez Alonso and colleagues' paper (Perez Alonso et al., 2024).

**S2. ANOVA table with all main effects and two-way interaction effects of the manipulated factors on ARI**

| factors | Df | Sum Sq | Mean Sq | F value | Pr(>F) | Partial η² |
| --- | --- | --- | --- | --- | --- | --- |
| $G$ | 1 | 4.869 | 4.869 | 216.768 | <2e-16** | 0.005 |
| $K$ | 1 | 172.945 | 172.945 | 7,698.941 | <2e-16** | 0.138 |
| Large $N_{g}$ | 1 | 46.328 | 46.328 | 2,062.383 | <2e-16** | 0.041 |
| Small $N_{g}$ | 1 | 104.755 | 104.755 | 4,663.320 | <2e-16** | 0.089 |
| Small groups prop | 4 | 869.400 | 217.350 | 9,675.681 | <2e-16** | 0.447 |
| $\beta$ | 2 | 822.804 | 411.402 | 18,314.216 | <2e-16** | 0.433 |
| Reliability | 1 | 12.121 | 12.121 | 539.586 | <2e-16** | 0.011 |
| Fixed/Random | 1 | 1,414.823 | 1,414.823 | 62,983.113 | <2e-16** | 0.568 |
| $G$ × $K$ | 1 | 0.164 | 0.164 | 7.294 | 0.007** | 0.000 |
| $G$ × Large $N_{g}$ | 1 | 0.225 | 0.225 | 10.029 | 0.002** | 0.000 |
| $G$ × Small $N_{g}$ | 1 | 0.000 | 0.000 | 0.012 | 0.912 | 0.000 |
| $G$ × Small groups prop | 4 | 0.186 | 0.047 | 2.071 | 0.082 | 0.000 |
| $G$ × $\beta$ | 2 | 0.284 | 0.142 | 6.325 | 0.002** | 0.000 |
| $G$ × Reliability | 1 | 0.004 | 0.004 | 0.177 | 0.674 | 0.000 |
| $G$ × Fixed/Random | 1 | 3.641 | 3.641 | 162.095 | <2e-16** | 0.003 |
| $K$ × Large $N_{g}$ | 1 | 8.401 | 8.401 | 373.982 | <2e-16** | 0.008 |
| $K$ × Small $N_{g}$ | 1 | 0.004 | 0.004 | 0.163 | 0.686 | 0.000 |
| $K$ × Small groups prop | 4 | 69.111 | 17.278 | 769.143 | <2e-16** | 0.060 |
| $K$ × $\beta$ | 2 | 29.227 | 14.614 | 650.549 | <2e-16** | 0.026 |
| $K$ × Reliability | 1 | 0.074 | 0.074 | 3.308 | 0.069 | 0.000 |
| $K$ × Fixed/Random | 1 | 3.969 | 3.969 | 176.672 | <2e-16** | 0.004 |
| Large $N_{g}$ × Small $N_{g}$ | 1 | 0.005 | 0.005 | 0.229 | 0.632 | 0.000 |
| Large $N_{g}$ × Small groups prop | 4 | 14.997 | 3.749 | 166.907 | <2e-16** | 0.014 |
| Large $N_{g}$ × $\beta$ | 2 | 24.101 | 12.051 | 536.447 | <2e-16** | 0.022 |
| Large $N_{g}$ × Reliability | 1 | 0.080 | 0.080 | 3.558 | 0.059 | 0.000 |
| Large $N_{g}$ × Fixed/Random | 1 | 8.522 | 8.522 | 379.387 | <2e-16** | 0.008 |
| Small $N_{g}$× Small groups prop | 4 | 111.147 | 27.787 | 1,236.977 | <2e-16** | 0.094 |
| Small $N_{g}$ × $\beta$ | 2 | 3.358 | 1.679 | 74.735 | <2e-16** | 0.003 |
| Small $N_{g}$ × Reliability | 1 | 0.093 | 0.093 | 4.136 | 0.042 | 0.000 |
| Small $N_{g}$ × Fixed/Random | 1 | 8.657 | 8.657 | 385.384 | <2e-16** | 0.008 |
| Small groups prop × $\beta$ | 8 | 181.503 | 22.688 | 1,009.989 | <2e-16** | 0.144 |
| Small groups prop × Reliability | 4 | 0.130 | 0.032 | 1.442 | 0.217 | 0.000 |
| Small groups prop × Fixed/Random | 4 | 95.498 | 23.875 | 1,062.816 | <2e-16** | 0.082 |
| $\beta$ × Reliability | 2 | 0.216 | 0.108 | 4.811 | 0.008** | 0.000 |
| $\beta$ × Fixed/Random | 2 | 102.716 | 51.358 | 2,286.280 | <2e-16** | 0.087 |
| Reliability × Fixed/Random | 1 | 12.464 | 12.464 | 554.846 | <2e-16** | 0.011 |

*Note. Signif. codes: 0.01 ‘**’*

**S3. Supplementary figures**

S3. Fig 1. The classification uncertainty for MixML-SEM in function of the within-group sample sizes for large and small groups, proportion of small groups, number of clusters, and size of regression parameters. Top: Fixed within-group samples. Bottom: Random within-group samples.


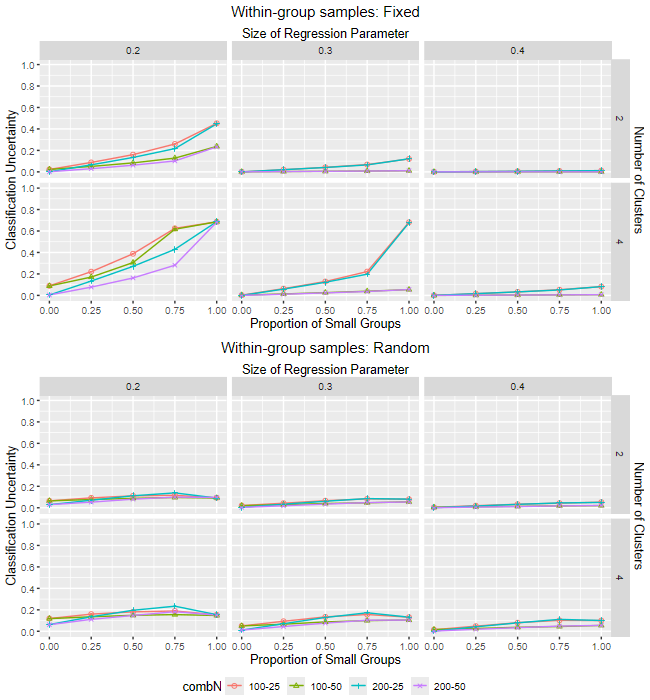


*Note.* “combN” refers to the combination of large and small groups.

S3. Fig 2. The ${RMSE}_{\beta2}$ for MixML-SEM in function of the within-group sample sizes for large and small groups, proportion of small groups, number of clusters, and size of regression parameters. Top: Fixed within-group samples. Bottom: Random within-group samples.


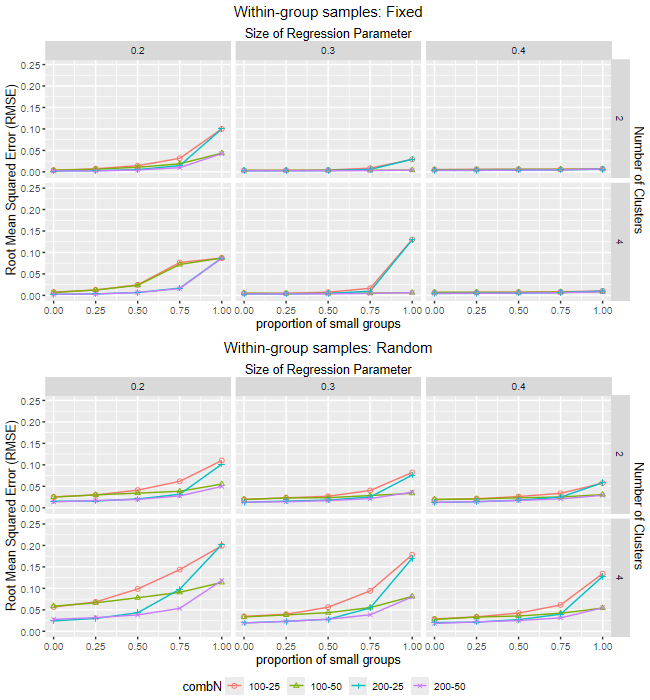


*Note.* “combN” refers to the combination of large and small groups.

S3. Fig 3. The ${RMSE}_{\beta3}$ for MixML-SEM in function of the within-group sample sizes for large and small groups, proportion of small groups, number of clusters, and size of regression parameters. Top: Fixed within-group samples. Bottom: Random within-group samples.


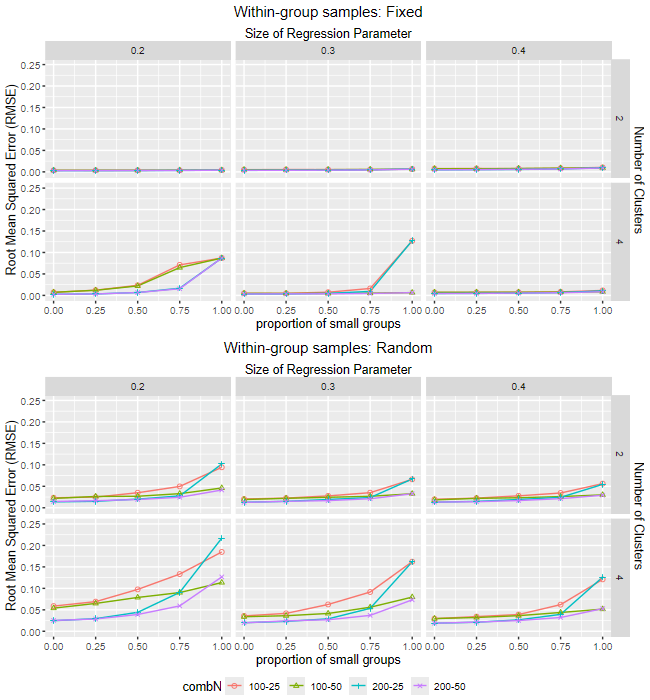


*Note.* “combN” refers to the combination of large and small groups.

S3. Fig 4. The ${RMSE}_{\beta4}$ for MixML-SEM in function of the within-group sample sizes for large and small groups, proportion of small groups, number of clusters, and size of regression parameters. Top: Fixed within-group samples. Bottom: Random within-group samples.
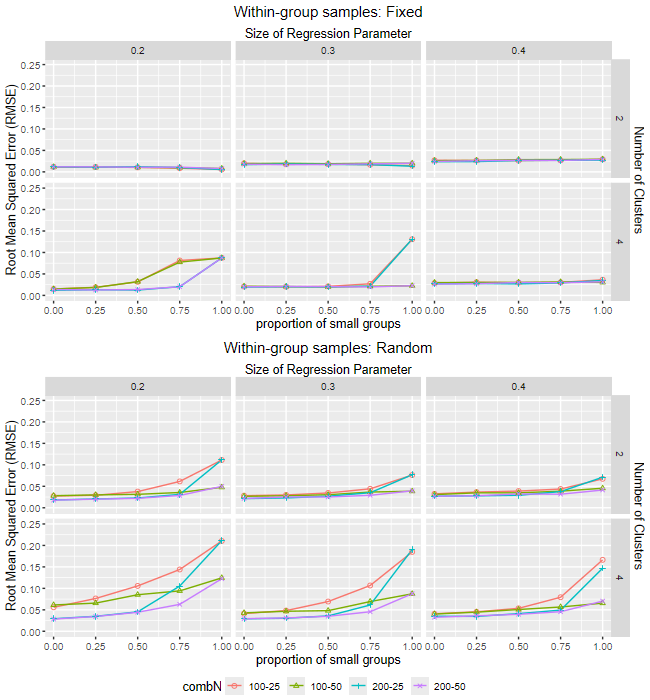


*Note.* “combN” refers to the combination of large and small groups.

**S4. Measurement invariance testing and final syntax for empirical application on social pressure to be happy and life satisfaction**

Before applying MixML-SEM to the empirical data, we evaluated measurement invariance for the SEHS and SWLS separately using ML-CFA in Mplus. We first estimated a ML-CFA model where all measurement parameters were set as random and examined their variances to identify those with the largest variance. Based on these variances, we estimated a series of ML-CFA models, each time adding one random measurement parameter in the order of the magnitude of their variances. These models were compared using the DIC, with lower DIC values indicating better model fit.

For SEHS, a better model fit was found after adding the residual covariances between certain SEHS items (specifically, items 1 and 2, and items 7 and 8) to the configural model, where all measurement parameters were set to be random. In this model, the unique variances, factor variances, and factor loadings of items 1, 6, and 9 had relatively large random effect variances. Therefore, we compared the model fit of the following models, where each one included random unique variances and factor variances: (1) no random loadings, (2) random loadings for item 1, (3) random loadings for items 1 and 9, and (4) random loadings for items 1, 6, and 9. The last model had the lowest DIC value, indicating the best balance between fit and complexity. The MI testing thus revealed three non-invariant factor loadings (item 1, 6, and 9). This was partially confirmed by running MG-CFA in *lavaan*, which suggested partial metric invariance with group-specific loadings for items 6 and 9. Specifically, item 1 (“Most people think that feeling Happy is a key indicator of success in life.”) strongly loaded on SEHS in a few countries (e.g., for Uganda, $\lambda=0.944$; for Senegal, $\lambda=0.943$), but presented relatively lower loadings in many other countries (e.g., for New Zealand, $\lambda=0.269$; for France, $\lambda=0.294$), indicating that it was a weaker indicator of SEHS in these countries. The mean loading of item 1 across all countries was 0.500 with a variance of 0.045. Item 6 (“Other people generally expect me to feel Happy.”) showed a strong loading for most countries while being weaker for China ($\lambda=0.332$). The mean loading was 0.807 with a variance of 0.029. Similarly, the loading of item 9 (“If I am honest, it is really important to me that others see me as someone who is always Happy.”) on SEHS was found to be strong for most countries but weaker for Senegal ($\lambda=0.432$) and Chile ($\lambda=0.502$). The mean loading was 0.735 with a variance of 0.040. For SWLS, a similar approach was used but no non-invariant loadings were found. Therefore, MixML-SEM was specified with no random loadings for SWLS and three random loadings for SEHS in addition to random unique variances and factor variances.

Below, we present the syntax for the final measurement model specified for the empirical example. Note that random unique variances cannot be specified for the items with residual covariances (SEHS: item 1, 2, 7 and 8) in Mplus. The code for the complete MI evaluation procedures can be found on osf (<https://osf.io/rtp78/>).

**SWLS**

MODEL ="

%within%

LS BY SWL_1 SWL_2 SWL_3 SWL_4 SWL_5; ! invariant factor loadings

logLS | LS; ! random factor variances

logSWL_1 | SWL_1; ! random unique variances

logSWL_2 | SWL_2;

logSWL_3 | SWL_3;

logSWL_4 | SWL_4;

logSWL_5 | SWL_5;

[SWL_1-SWL_5@0]

"

**SEHS**

MODEL ="

%within%

SEHS_latent BY SEHS_2* SEHS_3 SEHS_4 SEHS_5 SEHS_7 SEHS_8@1; ! invariant factor loadings

s1 | SEHS_latent BY SEHS_1; ! random factor loadings

s6 | SEHS_latent BY SEHS_6;

s9 | SEHS_latent BY SEHS_9;

logSEHS_latent |SEHS_latent; ! random factor variances

SEHS_7 WITH SEHS_8; ! residual covariances

SEHS_1 WITH SEHS_2;

logSEHS_3 |SEHS_3; ! random unique variances

logSEHS_4 |SEHS_4;

logSEHS_5 |SEHS_5;

logSEHS_6 |SEHS_6;

logSEHS_9 |SEHS_9;

[SEHS_1-SEHS_9@0]

"
